# MGF110-2L deletion prevents IFN-I and inflammatory response, resulting in partial attenuation and protection against virulent ASFV

**DOI:** 10.1128/jvi.00636-26

**Published:** 2026-06-26

**Authors:** Julia Gata-de-Benito, Marek Walczak, Lihong Liu, Gonzalo Vigara-Astillero, Krzesimir Szymankiewicz, Maciej Kochanowski, Jacek Żmudzki, Yolanda Revilla, Daniel Pérez-Núñez

**Affiliations:** 1 Microbes in Health and Welfare Department, Centro de Biología Molecular Severo Ochoa, CSIC-UAM, Madrid, Spain; 2 Department of Swine Diseases, National Veterinary Research Institute, Pulawy, Poland; 3 Department of Microbiology, Swedish Veterinary Agency, Uppsala, Sweden; 4 Department of Bacteriology and Bacterial Animal Diseases, National Veterinary Research Institute, Pulawy, Poland; Northwestern University Feinberg School of Medicine, Chicago, Illinois, USA

**Keywords:** IFN-I, inflammation, Arm/07/CBM/c2, LAV, vaccine, virulence, MGF110-2L, ASFV

## Abstract

African swine fever virus (ASFV) encodes numerous multigene family (MGF) proteins whose individual contributions to virulence and immune modulation remain poorly understood. Here, we characterize the role of MGF110-2L in shaping innate immunity and disease outcome *in vivo*. Deletion of MGF110-2L from the virulent Arm/07/CBM/c2 isolate markedly attenuated clinical disease and conferred complete protection against homologous challenge. In macrophages, the MGF110-2L-deficient recombinant induced enhanced type I interferon (IFN) responses, consistent with a role for MGF110-2L in antagonizing antiviral signaling. However, transcriptomic profiling of peripheral blood mononuclear cells revealed that the absence of MGF110-2L prevents the excessive IFN and inflammatory hyperactivation triggered by the parental virus *in vivo*, establishing a controlled antiviral program associated with reduced pathology. These findings identify MGF110-2L as a functional virulence factor that drives dysregulated innate immune responses during ASFV infection. Our work advances the molecular understanding of ASFV-host interactions and highlights the contribution of MGF110-2L protein to immune evasion and pathogenesis.

**IMPORTANCE:**

Finding safe and effective vaccines is essential to impair the spread of African swine fever virus (ASFV), responsible for the largest animal pandemic. Identifying ASFV virulence factors and understanding the mechanisms of pathogenesis and protection are crucial. By using RNAseq from *in vivo* peripheral blood mononuclear cells, together with immunological studies, we showed that MGF110-2L regulates type I IFN production and cytokine storm in pigs. Deletion of the MGF110-2L gene results in attenuation and induces protection against the parental virulent strain. These results support the induction of an alert immune state together with dampened systemic inflammation but preserved cytotoxic readiness in vaccinated animals. Altogether, our data shed light on the mechanisms underlying protection against ASFV and contribute to the development of new protective tools.

## INTRODUCTION

African swine fever virus (ASFV) is a dsDNA virus belonging to the *Asfarviridae* family (1) that exclusively infects monocytes and macrophages of domestic pigs and wild boars (2, 3), causing African swine fever (ASF), a disease endemic in Africa that causes up to 100% case fatality from virulent strains. In 2007, an outbreak of a virulent genotype II strain was detected in the Caucasus (4), and since then, ASFV has spread uncontrollably across Eastern Europe, currently reaching Western European countries such as Germany and Italy (5, 6), and very recently Spain (7). In addition, an outbreak was detected in China in 2018 (8), which led to the spread of the disease throughout this country and to neighboring countries such as Vietnam, Korea, or the Philippines, and recently to India and Indonesia (9–11). In 2021, ASFV outbreaks were detected in the Caribbean (12), threatening also the Americas. The ASFV currently represents the greatest threat to the global pig population, being present on four continents and in more than 60 countries, causing countless economic losses in the pig sector (13).

Most strains currently circulating in Europe and, to a significant extent, in Asia are highly virulent genotype II strains that cause acute infection in infected animals, triggering a cytokine storm, hemorrhages, and lymphopenia, leading to death within 7–12 days after infection (14–16). Furthermore, attenuated strains have also appeared naturally, both from the circulating genotype II and genotype I (17, 18), causing chronic disease and inflammation, without leading to the death of the animal. The significant differences in pathogenesis between infection with different strains have led to the study of the molecular mechanisms of ASFV virulence, which is one of the relevant topics in ASFV research. In recent years, it has become clear that one key way in which ASFV inhibits the host’s immune response, thereby increasing its virulence, is through its ability to control the innate immune response by controlling type I IFN (19). In particular, the cGAS/STING pathway is one of the main pathways controlled by the virus, and its ability to control this pathway largely determines the degree of virulence that the strain will have during infection within the animal (19, 20). Furthermore, the ASFV has been shown to impair the host’s capacity to express interferon-stimulated genes (ISG) by interfering with the JAK-STAT pathway through the activity of specific viral proteins (21).

The genome comparison between virulent and attenuated strains led to the identification of genes absent in the latter (22), which were thought to be involved in controlling the host’s immune system and, therefore, in virulence. These findings resulted in the development of live-attenuated vaccines (LAVs), which consist of deleting one or more virulence-related genes (23). LAVs remain the most realistic vaccine approach to date, as some prototypes provide 100% protection against homologous isolates (24–29), whereas other strategies, such as subunit or vector vaccines, achieve much lower protection levels, if any (30–32). However, safety issues associated with LAV prototypes (33–35) prevent their large-scale commercialization, particularly in non-endemic regions. Therefore, studying the molecular mechanisms and factors involved in ASFV virulence, alongside the protective mechanisms produced by LAVs, is crucial for developing safer and more effective tools against ASFV.

In this study, we identified MGF110-2L as a gene that controls type I IFN, both during infection and in ectopic experiments, by generating a recombinant virus designated Arm-ΔMGF110-2L. Moreover, the Arm-ΔMGF110-2L virus was shown to be partially attenuated *in vivo*, which reinforces the link between controlling the innate immune response and ASFV virulence. Finally, immunization provided total protection against a virulent challenge, establishing Arm-ΔMGF110-2L as a promising prototype for a baseline vaccine. To investigate the protective mechanisms induced by Arm-ΔMGF110-2L in more detail, we performed an RNA sequencing (RNA-seq) study, comparing vaccinated animals at 0 and 4 days post-challenge (dpc) with unvaccinated control animals. These analyses revealed that the vaccine induces a transcriptomic response similar to that observed in unvaccinated animals after ASFV infection. However, the expression levels of these specific genes were much lower than those in unvaccinated animals.

In conclusion, our study examined the protective mechanisms of the immune response induced by this LAV candidate, establishing a link between ASFV virulence mechanisms and the development of effective immune protection. Furthermore, the study presents Arm-ΔMGF110-2L as a promising platform for developing novel protective tools against ASFV.

## RESULTS

### Search for new ASFV virulence genes

**Fig 1:**
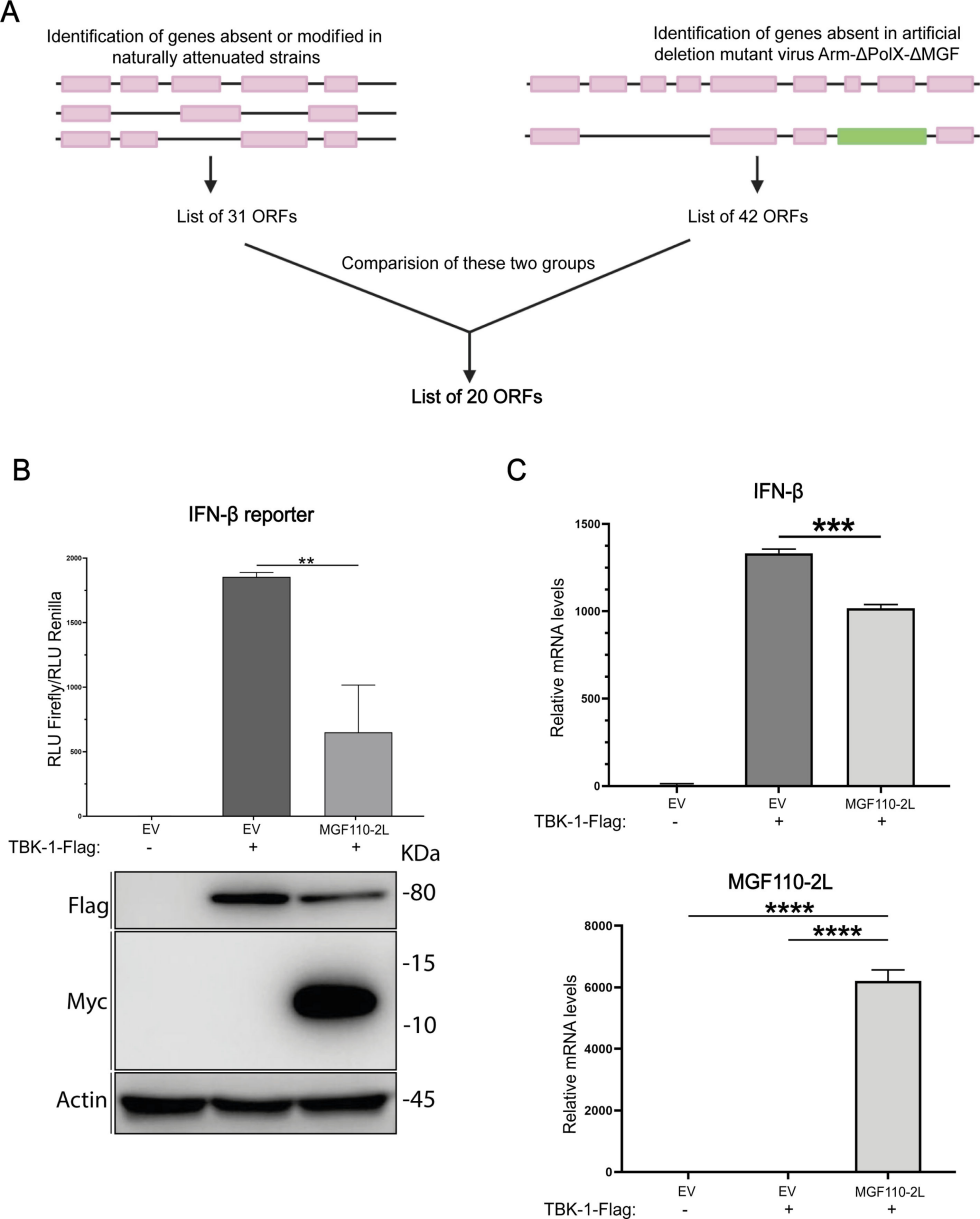
Ectopically expressed MGF110-2L inhibits IFN-β induction. (**A**) Outline of the *in silico* analysis to identify viral genes potentially involved in virulence. (**B**) HEK 293T cells were co-transfected with 2,000 ng/10⁶ cells of empty vector (pcDNA-3.1-myc) or viral gene (pcDNA-3.1-MGF110-2L-myc) together with TBK-1-Flag (100 ng/10⁶ cells), pIFNβ-Luc (100 ng/10⁶ cells), and pRTLK (50 ng/10⁶ cells); 24 h post-transfection (hpt), cells were lysed, and luciferase activity was measured using a dual-Luciferase kit (*n* = 3). Viral gene expression was detected by western blot using anti-myc (1/1,000), anti-FLAG (1/1,000), and anti-actin (1/6,000) antibodies. (**C**) HEK 293T were co-transfected with 2,000 ng/10⁶ cells of empty vector (pcDNA-3.1-myc) or pcDNA-MGF110-2L-myc together with TBK-1-Flag (100 ng/10⁶ cells). Cells were harvested at 24 hpt and analyzed using RT-qPCR for IFN-β (upper panel) and viral MGF110-2L (**C**) for mRNA levels (*n* = 3). Data were statistically analyzed by one-way ANOVA (**P < 0.01; ***P < 0.001; ****P < 0.0001).

In order to identify new ASFV genes involved in virulence, an *in silico* analysis identifying genes that were either absent or modified in naturally attenuated or adapted strains and present in virulent strains of genotype I and genotype II were employed. For that, we performed a comparative assay of the proteome of eight different ASFV strains (Table S1) by using BlastP software, and the output was analyzed for protein clusters, containing similar proteins between the strains, using the Markov Cluster Algorithm (MCL) ( https://doi.org/10.1093/nar/30.7.1575). The algorithm generated a total of 165 protein clusters, which were afterward classified according to the number of proteins included. We discarded clusters containing eight proteins (that corresponded to a protein per strain) from the analysis, and the remaining 56 clusters were analyzed: nine clusters included more than eight proteins per cluster, whereas 47 clusters contained fewer than eight proteins per cluster. Each of these clusters was individually analyzed, searching for proteins that were absent or modified in attenuated or adapted strains, revealing 31 genes potentially involved in ASFV virulence. Conversely, we employed information derived from the Arm-ΔPolX-ΔMGF, an attenuated mutant available in the lab (36), which spontaneously emerged during the generation of the recombinant virus Arm-ΔPolX, and lacked 42 genes. An assessment of the genes coming from comparison between these two experimental analyses resulted in a list of 20 genes considered to be potentially involved in virulence (Fig. 1A).

In order to analyze whether any of these genes are involved in the control of type I IFN production, one of the main host factors regulating ASFV virulence, genes were separately cloned into a pcDNA-myc expression vector and transfected in HEK-293T cells, although not all of them were cloned. Furthermore, in some cases, these proteins were not expressed ectopically. When possible, their expression was then detected by western blot using an anti-myc antibody, and the genes that were successfully expressed were selected (see as an example Fig. S1).

### Ectopic expression of MGF110-2L inhibits IFN-β production

Next, we screened whether any of the genes identified explained above could control IFN-β production. By using a specific luciferase assay, the MGF110-2L gene was found to be a potential IFN-I-immunomodulatory gene (Fig. 1B). The expression of TBK1, a component of the cGAS/STING pathway involved in IFN-β production, resulted in an increase in luciferase activity, counteracted by MGF110-2L expression. To confirm this observation, the amount of IFN-β-specific mRNA that was repressed by MGF110-2L was assessed by RT-qPCR. For that, HEK-293T cells transfected with TBK1-FLAG, with and without MGF110-2L-myc, were tested for IFN-β production. As demonstrated in Fig. 1C, the expression of MGF110-2L efficiently counteracted the production of IFN-β mRNA induced by TBK1, thereby confirming the results of the luciferase assay (Fig. 1B). From these results, we conclude that MGF110-2L regulates the IFN-β production in ectopic conditions.

### The deletion of the MGF110-2L gene does not affect ASFV *in vitro* growth

**Fig 2:**
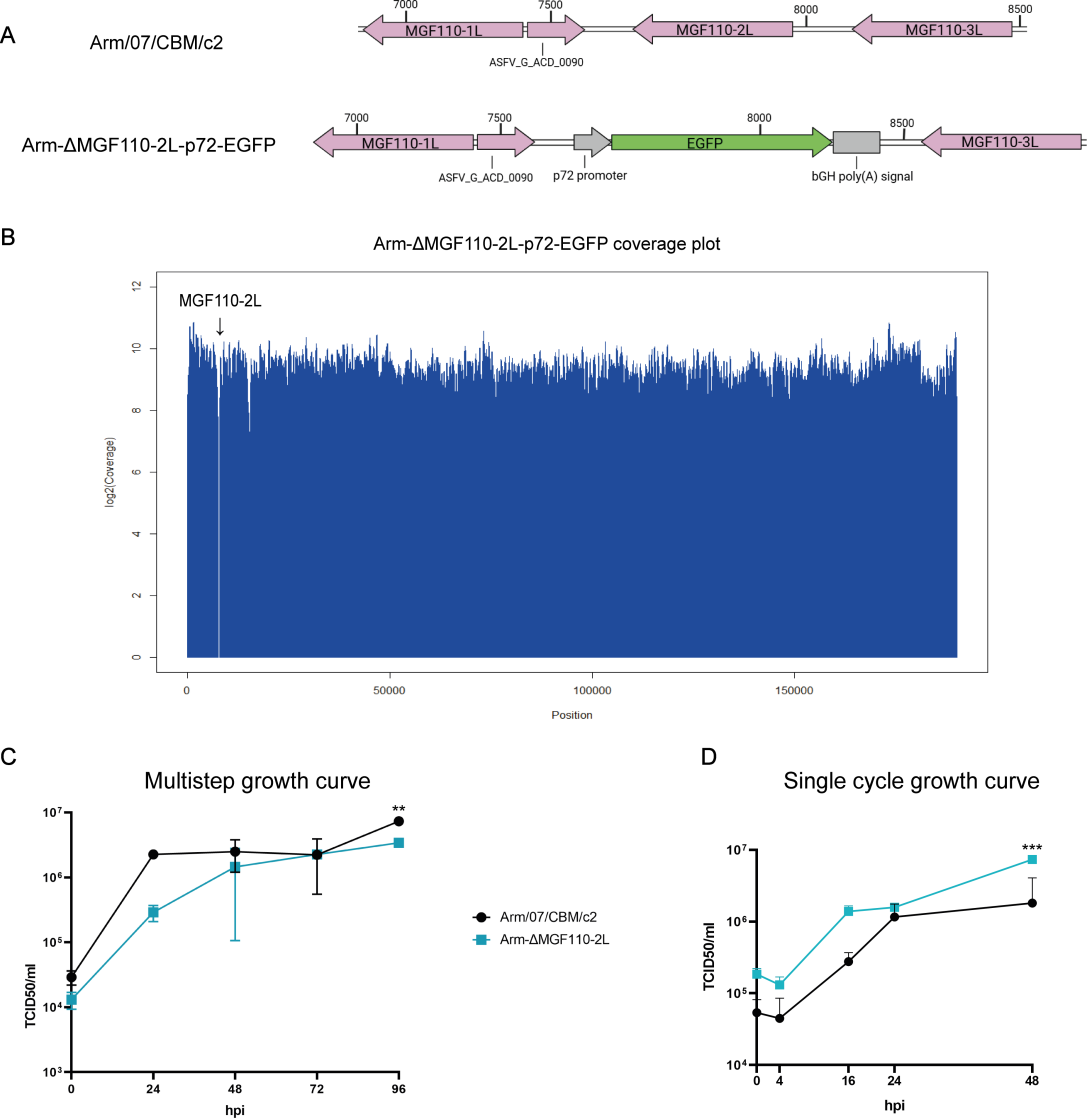
Generation, sequencing, and growth kinetics of Arm-ΔMGF110-2L. (**A**) Schematic representation of the replacement of the MGF110-2L by the EGFP cassette under the viral p72 promoter by CRISPR/Cas9 technology. (**B**) Coverage plot of Arm-ΔMGF110-2L sequenced by Illumina. MGF110-2L gene deletion is shown. (**C**) Multistep growth curve comparison of Arm-ΔMGF110-2L and its virulent parental, Arm/07/CBM/c2. PAMs were infected with an MOI of 0.5 of each virus. Infections were collected at 0, 24, 48, 72, and 96 hpi, and viruses were titrated by TCID50/mL. (**D**) Single-cycle growth curve comparison of Arm-ΔMGF110-2L and its virulent parental strain, Arm/07/CBM/c2. PAMs were infected with an MOI of five for each virus. Samples were collected at 0, 4, 16, 24, and 48 hpi, and the viruses were titrated by TCID₅₀/mL (*n* = 2). (**C and D**) Arm/07/CBM/c2 is represented in black and Arm-ΔMGF110-2L in blue. The data were statistically analyzed using ordinary two-way ANOVA (**P < 0.01; ***P < 0.001).

To study the effect of MGF110-2L during ASFV infection, we generated a recombinant virus by deleting the MGF110-2L gene from the virulent parental strain Arm/07/CBM/c2 (LR812933.1), resulting in the Arm-ΔMGF110-2L mutant. We used our well-established CRISPR/Cas9 technology (28, 37) to replace the MGF110-2L gene with an EGFP marker expression cassette under the B646L viral strong promoter, which encodes the p72 protein (Fig. 2A). The recombinant virus was obtained, purified by successive rounds of plaque isolation, and DNA was extracted from the extracellular viral particles. Illumina platform sequencing confirmed the absence of the MGF110-2L gene (Fig. 2B). Illumina reads were *de novo*-assembled, obtaining a viral genome of 193,174 base pairs. The alignment of the viral genome against parental Arm/07/CBM/c2 and the predicted *in silico* assembly of Arm-ΔMGF110-2L-GFP genomes showed the absence of the MGF-110-2L gene and its substitution by the expression cassette of EGFP as designed. These data indicate that MGF110-2L is not essential for ASFV viability. Variant calling analysis revealed very few additional variations, as shown in . Although there is an insertion corresponding to the MGF110-14L gene, which falls within a polyC region. This region has been shown to be difficult to sequence, with variations being usually observed in different ASFV isolates or mutants; hence, the observed variation is probably the result of the sequencing process. Another insertion was found in an intergenic region (at position 15,444), and a third variation was observed in the EP424R gene, producing the amino acid change Y307H. Beyond these minor variations, the only difference observed compared to the Arm/07/CBM/c2 virus is the absence of the MGF110-2L gene. To confirm that the insertion of the EGFP cassette did not affect the expression of genes neighboring the deleted gene, we verified the expression of the MGF110-1L and MGF110-3L genes located downstream and upstream of MGF110-2L, respectively, using RT-qPCR. As shown in Fig. S2, at 4 hpi, the expression of MGF110-1L and MGF110-3L did not change during infection with Arm-ΔMGF110-2L compared to the parental virus.

Once the virus has been genetically characterized, we performed multi-step and single-step growth curves in porcine alveolar macrophages (PAMs) comparing Arm-ΔMGF110-2L to Arm/07/CBM/c2 to assess whether the MGF110-2L gene could affect virus growth *in vitro*. As shown in Fig. 2C, at a low multiplicity of infection (MOI), although Arm-ΔMGF110-2L exhibits a slight delay in growth compared to the parental virus at early times, no significant differences are observed between the two viruses at the multistep growth curve, except at 96 hpi, where Arm/07/CBM/c2 WT grows slightly higher than the recombinant virus. Accordingly, there also appear to be no significant differences in the growth of the two viruses at a high MOI, although a slightly higher growth rate is observed for the mutant in the single-cycle growth curve, only significant at 48 hpi (Fig. 2D). These data suggest that MGF110-2L should not be involved in *in vitro* growth.

### The ASFV viral gene MGF110-2L controls IFN-β production during ASFV infection

**Fig 3:**
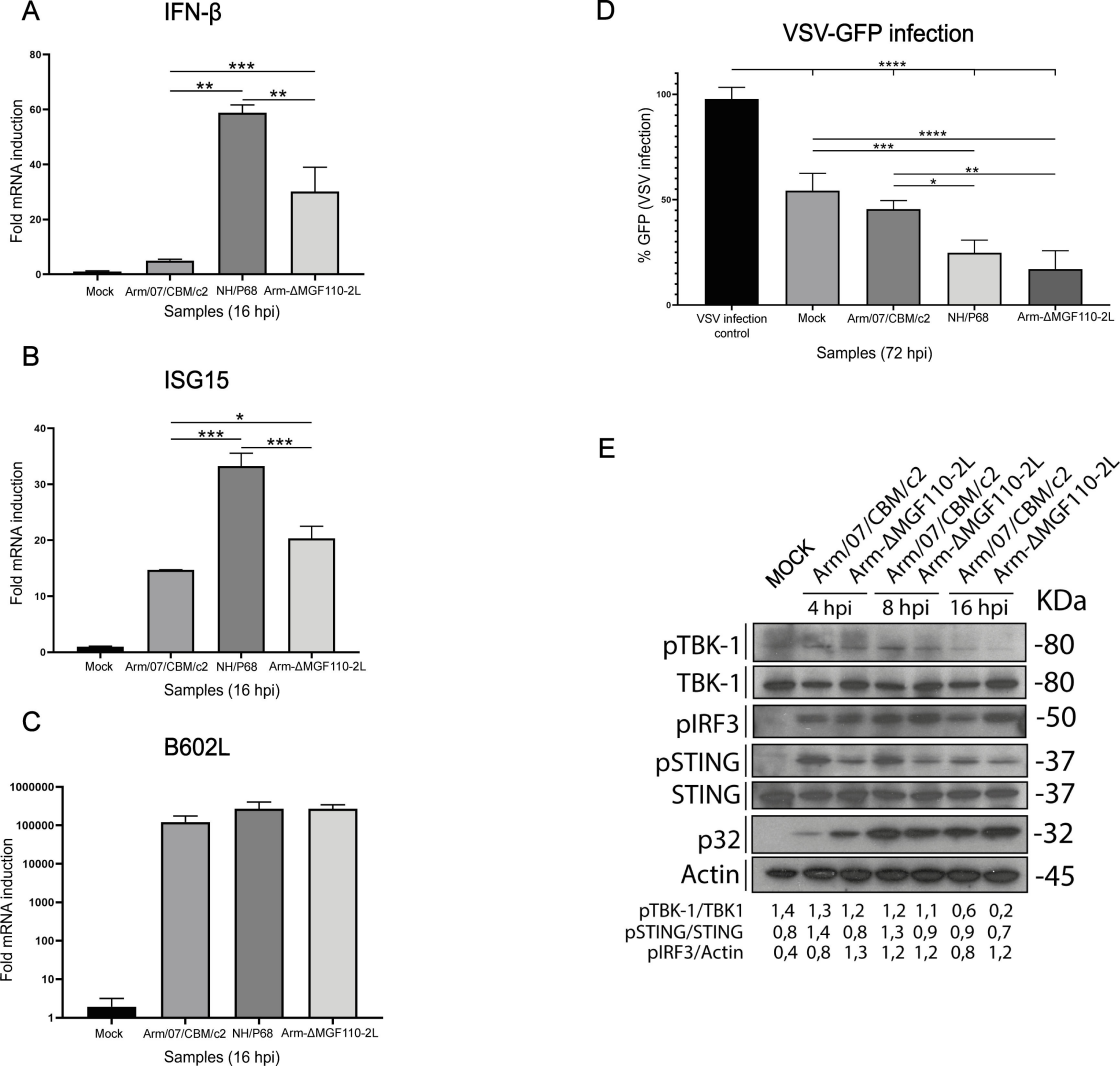
Arm-ΔMGF110-2L infection induces IFN-β expression. PAM cells were infected with Arm/07/CBM/c2, NH/P68, or Arm-ΔMGF110-2L with an MOI = 2 or not infected (mock). At 16 hpi, cells were collected and analyzed using RT-qPCR for IFN-β (**A**), ISG15 (**B**), and viral B602L (**C**) mRNA levels (Mean ± SD; *n* = 3). Data were statistically analyzed by one-way ANOVA (ns, *P* > 0.05; *P < 0.05; ***P < 0.001). (**D**) HeLa cells were seeded (5,000 cells/well) and incubated with inactivated supernatants from 72-h infections Arm/07/CBM/c2, NH/P68, Arm-ΔMGF110-2L (MOI = 0.5), or mock-infected. After 24 h of incubation, the supernatant was removed, and the cells were infected with VSV-GFP (MOI = 2). A control sample that had not been incubated with the supernatant was used as a positive infection control. Fluorescence intensity (488 nm) was measured at 16 hpi using a ClarioSTAR plate reader (Isogen Life Sciences) to calculate the percentage of infected cells. Data were statistically analyzed by one-way ANOVA (*P < 0.05; **P < 0.01; ***P < 0.001; ****P < 0.0001). (**E**) PAMs were infected with either Arm-ΔMGF110-2L or Arm/07/CBM/c2 at MOI = 2. Samples were collected at 4, 8, and 16 hpi and lysed in radioimmunoprecipitation assay (RIPA), and total expression and phosphorylation of the indicated proteins were detected by western blot by using anti-pTBK1 (1/1,000), anti-TBK1 (1/1,000), anti-pIRF3 (1/1,000), anti-pSTING (1/1,000), anti-STING (1/1,000), anti-p32 (1/3,000), and anti-Actin (1/1,000).

To further verify whether MGF110-2L controls IFN-β production during ASFV infection, we analyzed the amount of IFN-β mRNA produced during Arm-ΔMGF110-2L infection in PAMs, comparing it to the amount produced during infection with the virulent parental strain Arm/07/CBM/c2 and to the attenuated strain NH/P68 using RT-qPCR. As shown in Fig. 3A, Arm-ΔMGF110-2L infection produces significantly higher IFN-β than Arm/07/CBM/c2 infection. It is noteworthy that a significantly higher production of IFN-β mRNA is observed in PAMs infected with NH/P68. In addition, we analyzed the expression of the IFN-stimulated gene ISG15. Consistent with IFN-β expression, a significant increase in ISG15 expression was also observed in PAMs infected with either Arm-ΔMGF110-2L or NH/P68 compared to those infected with the virulent parental strain (Fig. 3B), while the infection level was similar, as observed from the expression of the B602L viral gene, used as a reference (Fig. 3C).

Furthermore, we assessed type I IFN activity using a vesicular stomatitis virus-green fluorescent protein (VSV-GFP)-based bioassay. In this assay, type I produced IFN inhibits VSV replication and reduces GFP expression, as previously described (38). We previously assessed that the amount of IFN-β in the supernatant was inversely proportional to the detected GFP intensity, as determined using a standard with known amounts of IFN-β (Fig. S3A). Additionally, ASFV-infected supernatants must be inactivated to prevent the presence of extracellular virus. This was previously verified by incubating the supernatants with COS-1 cells and confirming the absence of infected cells via flow cytometry (FACS), compared to non-inactivated sera (Fig. S3B).

After setting up the experimental conditions, PAMs were infected with the virulent strain Arm/07/CBM/c2, the recombinant virus Arm-ΔMGF110-2L, or the attenuated strain NH/P68, all at an MOI of 0.5. At 72 hpi, the cell culture supernatant was collected and inactivated. A non-infected supernatant was used as a positive control, and the corresponding supernatants from the different ASFV infections were then incubated with HeLa cells for 24 h, after which the cells were infected with VSV-GFP (MOI = 2) for a further 24 h. The intensity of GFP was then measured as a sign of VSV infection. As shown in Fig. 3D, consistent with the mRNA expression data (Fig. 3A), a significantly higher percentage of VSV-GFP infection was detected in cells incubated with the PAM-infected Arm/07/CBM/c2 supernatant than with the PAM-infected NH/P68 or Arm-ΔMGF110-2L supernatant (Fig. 3D). These results show a higher presence of functional type I IFN in cells infected with Arm-ΔMGF110-2L or NH/P68 than in cells infected with Arm/07/CBM/c2. Altogether, these data indicate that MGF110-2L controls IFN-β production, which is consistent with previous results obtained through ectopic expression (Fig. 1).

Production of IFN-β is a hallmark of ASFV virulence. As we have previously demonstrated, and as has been validated in several studies, IFN-β production *in vitro* is inhibited by infection with virulent strains, while there is a significant increase in its production during infection with attenuated strains (19, 28, 37). In view of the results in Fig. 3, we could deduce that Arm-ΔMGF110-2L may exhibit an attenuated phenotype, although IFN-β production, at least at the mRNA level, was significantly higher during NH/P68 infection.

We have previously shown that one of the main pathways of type I IFN production controlled by ASFV is the cGAS/STING pathway (19); hence, we investigated whether MGF110-2L exerted IFN-I control through this pathway. To achieve this, we analyzed the phosphorylation of STING, TBK1, and IRF3 at different times post-infection using specific antibodies by western blot to assess their activation. However, no increase in the phosphorylation of any of these proteins was observed during Arm-ΔMGF110-2L infection compared to infection with the virulent parental Arm/07/CBM/c2, although it cannot be ruled out that there might be some residual activity of this route modulated by MGF110-2L gene (Fig. 3D). These data suggest that the activation of this pathway during ASFV infection is not controlled by MGF110-2L.

Overall, MGF110-2L appears to be involved in controlling IFN-β during ASFV infection, albeit not through the cGAS-STING pathway, indicating that MGF110-2L could be a virulence gene.

### Arm-ΔMGF110-2L is partially attenuated *in vivo* and induces protection against the virulent strain Arm/07/CBM/c2

**Fig 4:**
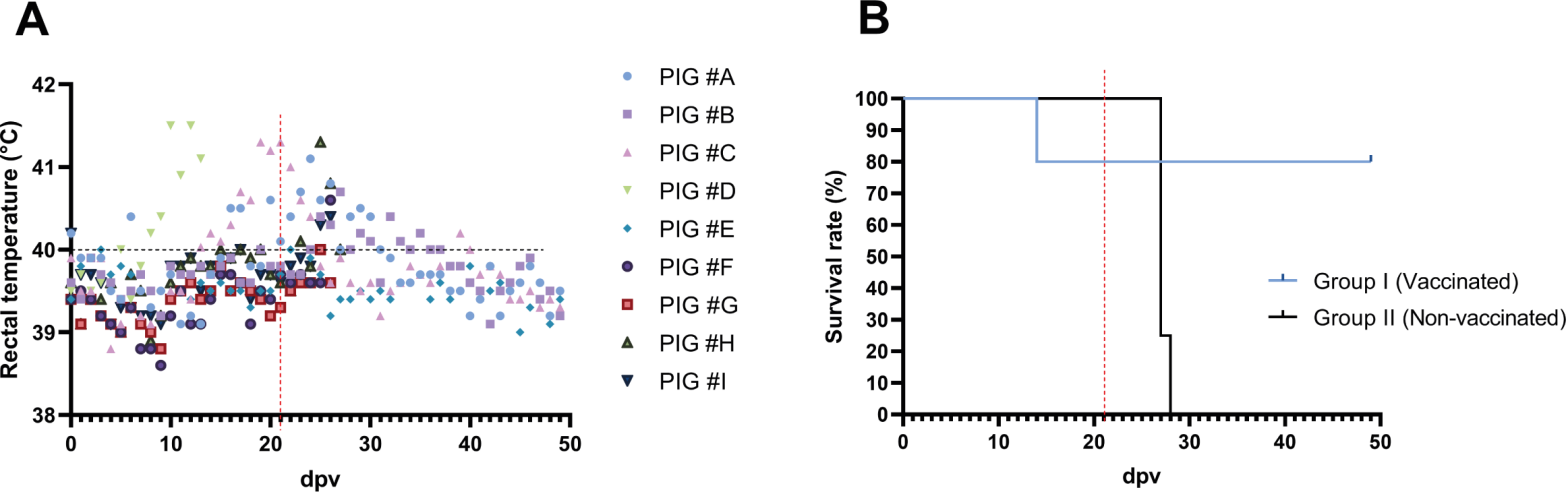
Arm-ΔMGF110-2L is partially attenuated *in vivo* and confers protection against the virulent Arm/07/CBM/c2 strain challenge. (**A**) Individual rectal temperature of vaccinated animals with Arm-ΔMGF110-2L (pig #A-#E) and non-vaccinated animals (pig #F-#I) measured from 0 to 49 dpv. (**B**) Percentage of survival of vaccinated animals immunized with Arm-ΔMGF110-2L (Δ2L group, blue line) or not vaccinated (control group, red line) at 49 dpv (28 dpc). Error bars indicate standard deviation. The black dashed line indicated the fever threshold (40°C). The red-dotted line indicates the challenge day (21 dpv).

To ascertain the possible attenuation of Arm-ΔMGF110-2L, together with its potential to induce protection against a virulent challenge, we conducted an *in vivo* trial in domestic pigs. To this end, a total of nine pigs were divided into two experimental groups, group Δ2L (*n* = 5, pigs A–E) and the control group (*n* = 4, pigs F–I). The animals in the Δ2L group were immunized intramuscularly (IM) with a dose of 10^3^ TCID50 per animal and monitored together with the unimmunized animals in the control group for 21 days, when all animals were challenged with an IM dose of Arm/07/CBM/c2 (10^1^ TCID50 per animal) and monitored for another 28 days. Fever followed by apathy was observed before challenge among all vaccinated animals (Δ2L group) except one pig (pig E), and a minimal incubation period was estimated as 8 days (Fig. 4A). At 14 days post-vaccination (dpv), one animal (pig D, Δ2L group) was euthanized due to reaching humane endpoint criteria, presenting recumbency and hypothermia, together with other ASF symptoms. It should be noted that this immunized animal was euthanized before the challenge took place, and that is the reason why the non-immunized controls survived longer at this stage. No clinical abnormalities were observed in the control group before the challenge. Overall, 4 out of 5 vaccinated animals survived and only one (pig #C) had a temperature over 40°C on the day of the challenge. Virulent challenge was then performed on all animals at 21 dpv. All the remaining vaccinated animals (4/5) remained healthy until the end of the experiment (49 dpv and 28 dpc), showing a decreasing tendency in recorded rectal temperature. In contrast, fever and apathy were recorded, leading to the death of all non-vaccinated animals, which were found dead between 27 dpv (6 dpc) and 28 dpv (7 dpc) (Fig. 4A and B).

**Fig 5:**
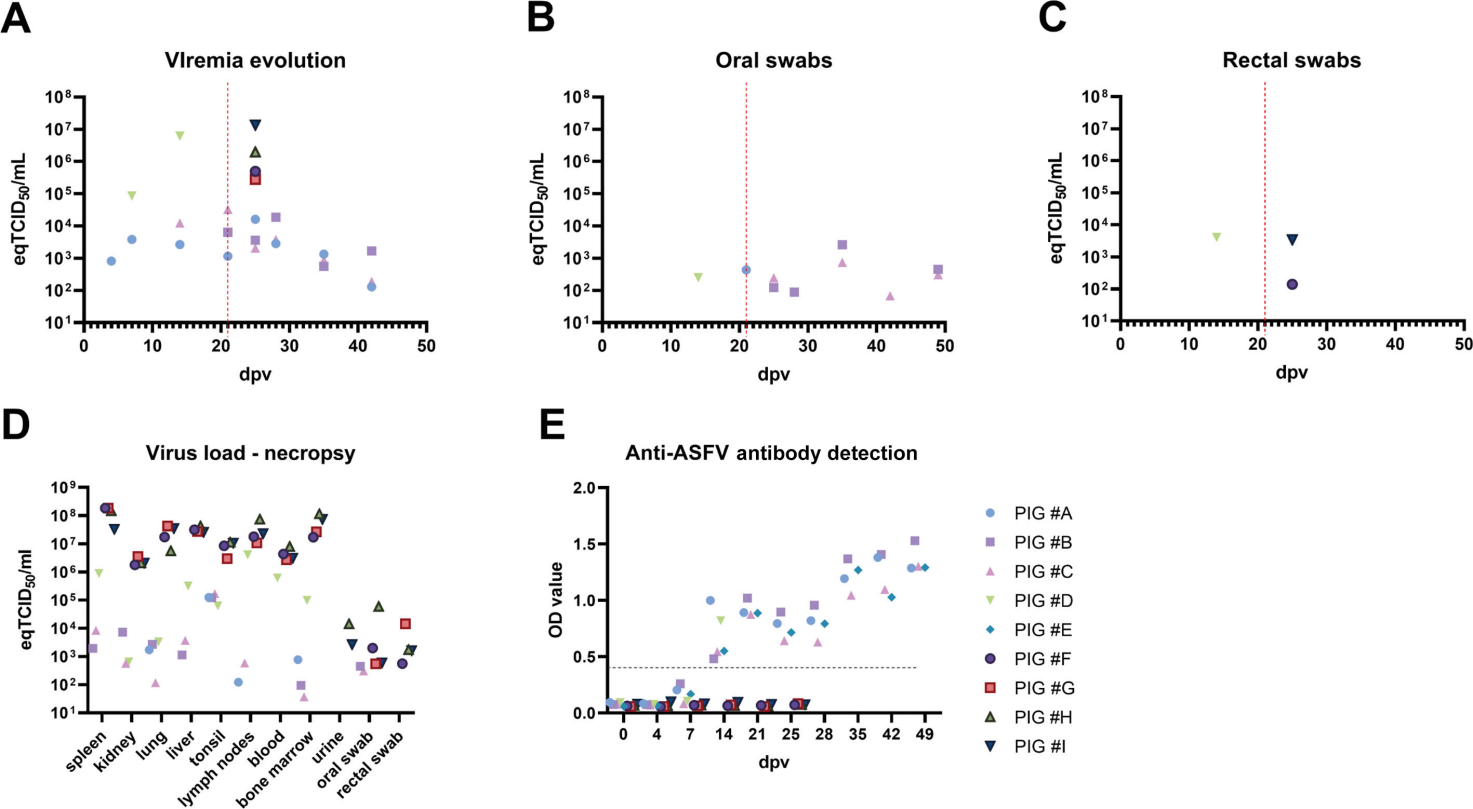
Viremia evolution, shedding pattern, virus load in tissues, and antibodies detection from control or vaccinated animals with Arm-ΔMGF110-2L. (**A**) Viremia evolution during the experiment (mean Ct values). (**B**) Shedding pattern in oral swabs. (**C**) Shedding pattern in rectal swabs. (**D**) Viral load in internal organs and chosen samples collected during necropsy. (**E**) Detection of antibodies in sera from control or animals vaccinated with Arm-ΔMGF110-2L. Optical density (OD) values of anti-ASFV antibody detection. Red-dashed line represents the challenge time point. Black dashed line indicates the mean negative cutoff threshold (ELISA). Dpv, days post-vaccination.

On the other hand, viremia was analyzed by qPCR in both vaccinated and non-vaccinated animals before and after challenge. At 4 dpv, viral DNA started to be detectable in the blood of one vaccinated animal, and until the challenge day (21 dpv), viremia was present in 4/5 animals from vaccinated animals (Δ2L group) (Fig. 5A). Nearly all animals (except pig E) in both groups tested positive for viral DNA by qPCR at 25 days post-vaccination (4 dpc). To determine whether the viremia observed after the challenge was due to the vaccine virus or the challenge virus, we performed conventional PCR to distinguish between the two (Fig. S4). At 4 dpc, only the recombinant virus was detected, and not the virulent challenge virus, indicating that, despite exposure to the virus, replication of the wild-type virus was inhibited in this group (Fig. S4A). In contrast, the genetic material of ASFV was not detected in the blood of vaccinated, survivor animals at the end of the experiment (49 dpv, 28 dpc) (data not shown).

Regarding the detection of ASFV genetic material in oral and rectal swabs, the ASFV genetic material was detected in some vaccinated animals before the challenge; however, higher detection levels were observed after the challenge and were detectable until the end of the experiment in oral swabs of pig B and pig C (Fig. 5B and C).

Samples of blood, urine, and oral swabs collected from surviving animals during necropsy showed to be negative for ASFV genetic material. Lower viral load was noticeable in all samples collected from Δ2L group during necropsy, out of which some internal organs (i.e., spleen, kidney, liver, and tonsil) showed reduced viral load compared to animals from the control group (Fig. 5D). PCR-based differentiation revealed that in the tonsils of vaccinated animals (3/4), only the recombinant virus persisted at the time of necropsy (Fig. S4B). No ASFV genetic material was detected in any samples collected during necropsy from pig E (Δ2L group).

During necropsy, typical ASF macroscopic lesions (i.e., splenomegaly, hemorrhagic lymphadenitis, and hyperemic tonsils) accompanied by a high amount of exudative fluid found in the abdominal cavity were found in all animals from the control group. In contrast, milder lesions were found in animals that survived the challenge from the Δ2L group, although enlargement of submandibular lymph nodes was noticeable. Only the euthanized vaccinated animal (pig D, euthanasia at 14 dpi) presented typical subacute ASF lesions such as lymphadenitis, splenomegaly, and hemorrhages in the heart, lungs, and petechiae in the kidneys and intestines (Table 1).

**TABLE 1:** Frequency of lesions observed in necropsy

Lesion	Δ2L^*b*^ (*N* = 5)	Frequency (%)	Control^*b*^ (*N* = 4)	Frequency (%)
Splenomegaly	1	20	4	100
Hyperemia and/or enlargement of lymph nodes^*a*^	3	60	4	100
Abdomen exudative fluid	1	20	4	100
Hyperemia of tonsil	1	20	3	75
Petechiae in kidneys	1	20	0	0
Pleural exudative fluid	0	0	1	25
Hyperaemia of lungs	2	40	1	25
Nasal discharge	0	0	0	0
Pericardial exudative fluid	1	20	1	25

^*a*^ Submandibular, gastro-hepatic, or mesenteric.

^*b*^ Number of animals with detected lesions.

### Vaccination with Arm-ΔMGF110-2L activates humoral response

Vaccination with Arm-ΔMGF110-2L causes a strong humoral immune response. Increased optical density (OD) values were found in animals from the Δ2L group started at 7 dpv and at 21 dpv, and all remained vaccinated pigs (4/5) were found to be seropositive. Mean OD values at 21 dpv (day of challenge) were estimated as 0.92 (±0.07) in the Δ2L group. These values increased after the challenge until the end of the experiment. In contrast, all challenged animals from the control group were found to be seronegative (Fig. 5E).

### Transcriptomic footprint of vaccination

**Fig 6:**
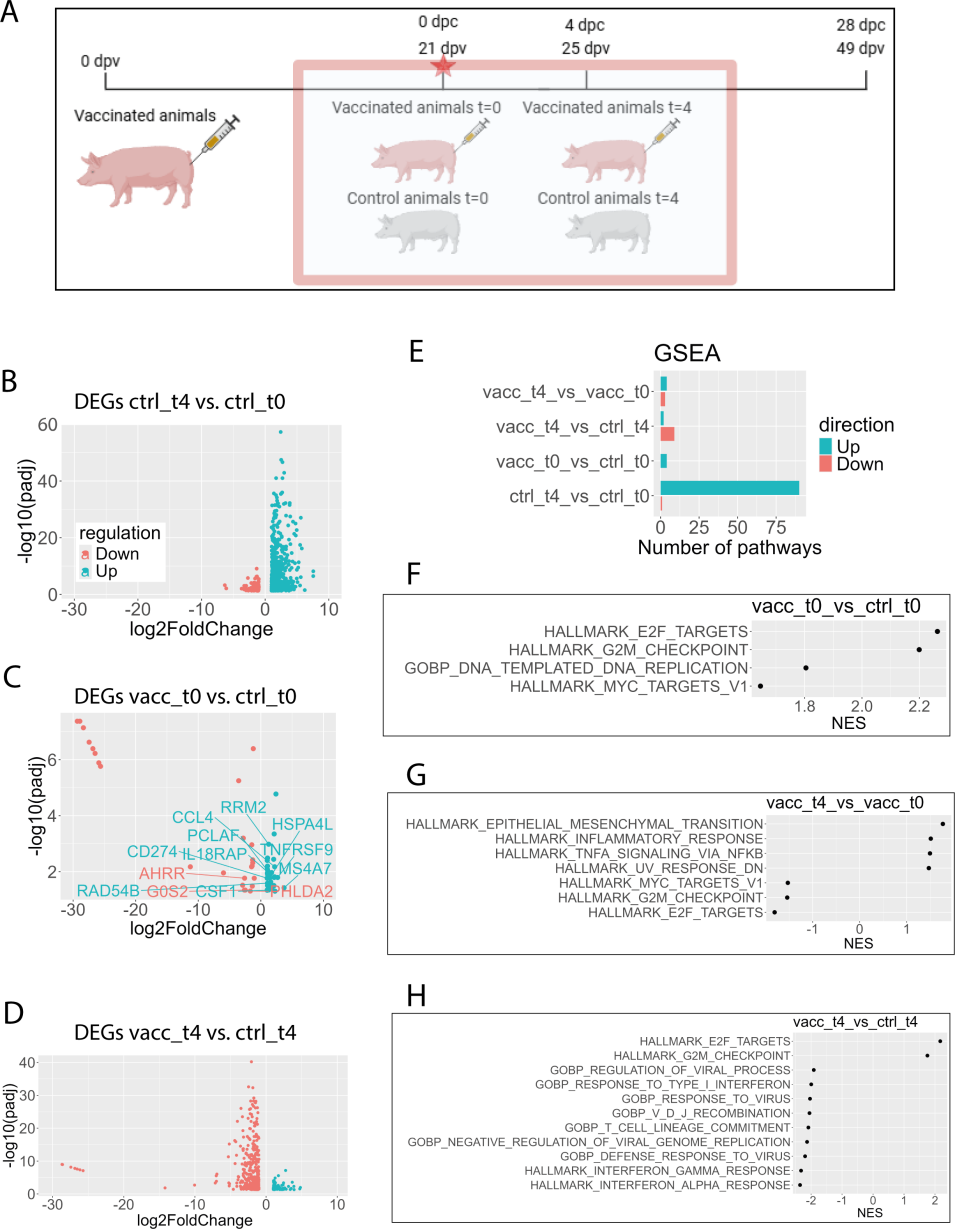
Transcriptomic impact of infection and vaccination in PBMCs revealed by RNA-seq**.** (**A**) Schematic overview of the experimental design indicating time points and sample collection strategy across vaccinated and unvaccinated groups. (**B–D**) Volcano plots representing differential gene expression for the indicated pairwise comparisons. The X-axis denotes the log2 fold change (log2FC), while the Y-axis indicates statistical significance (−log10 adjusted *P*-value). Red and blue dots highlight significantly upregulated and downregulated genes, respectively (padj < 0.05). (E–H) GSEA of transcriptomic responses in PBMCs across four experimental comparisons. (**E**) Global visualization of enriched pathways in each condition, sorted by normalized enrichment score (NES). Color bars indicate the direction and magnitude of enrichment (NES). (F–H) Representative significantly enriched pathways identified by GSEA in the following comparisons. (**F**) Ctrl_t4 vs. Ctrl_t0. (**G**) Vacc_t4 vs. Vacc_t0. and (**H**) Vacc_t4 vs. Ctrl_t4.

To identify the transcriptomic footprint of the effects of vaccination in animals both prior to and during the challenge, we performed an RNA-seq study of peripheral blood mononuclear cells (PBMCs) isolated from whole blood samples taken from control and vaccinated animals at 0 (21 dpv) or 4 days post-challenge (0 vs. 4 dpc) as shown in Fig. 6A. Although vaccination with a LAV can cause symptoms that are consistent with a subacute infection, as demonstrated by the increase in body temperature observed in animal #C, the remaining vaccinated animals did not exhibit any symptoms of infection overall. We therefore consider these effects to be a signature of vaccination, distinct from the effects of an acute infection, as discussed below.

Overall, principal component analysis (PCA) of the RNA-seq data set revealed distinct separation of samples based on treatment and time post-infection (Fig. S5A). Notably, samples from the control group at 4 days post-challenge (ctrl_t4) were categorized separately from the rest of the groups, indicating a unique transcriptomic profile linked to the progression of infection in the absence of immunity. In contrast, samples from the vaccinated group at 4 dpc (vacc_t4) remained closer to those from the vaccinated at 0 dpc (vacc_t0) as well as control at 0 dpc (ctrl_t0), suggesting that vaccination did not induce substantial transcriptomic shifts and that the challenge caused minimal changes in the vaccinated group. This pattern is consistent with the view that vaccination limits infection-induced transcriptional perturbations, thereby supporting a more regulated immune response that provides protection.

To analyze the transcriptional effects associated with vaccination and post-challenge viral infection, differentially expressed genes (DEGs) were identified in each experimental comparison as shown in a volcano plot (Fig. 6B through D). A functional enrichment study was also performed to identify the corresponding pathways involved in each condition and characterize the predominant molecular events (Fig. 6E through H and ).

First, the ctrl_t4 vs. ctrl_t0 condition was analyzed, highlighting the genes modulated during ASFV infection in the absence of a vaccine (Fig. 6B). A notable number of DEGs were observed, which is consistent with an intense immune response to ASFV infection in the animal, possibly involving inflammation or dysregulation. Most of these genes were found to be upregulated (150 vs. 105 with log2FC > 1 and padj <0.05), which may indicate the activation of immune defense pathways, a response to cell damage, or systemic inflammation. The classical antiviral genes, such as IFIT1/3, ISG15, OAS1, and MX1, were strongly upregulated, indicating the induction of type I interferon response. Upregulation of CXCL10, CCL2, and IL1B suggested the recruitment and activation of immune cells, while increased expression of SIGLEC1, FCGR1A, CD14, and TREM1 reflected the activation of circulating monocytes and dendritic cells, consistent with a systemic innate immune response in unvaccinated pigs. Although fewer in number, the downregulated genes (e.g., ABCA6, MMP8, and COBL) could be involved in functions that are suppressed during infection, such as cell metabolism, tissue repair, or homeostatic maintenance. Functional enrichment analysis using Gene Set Enrichment Analysis (GSEA) identified significantly activated pathways at the transcriptomic level (Fig. 6E and Fig. S5B). In particular, classic antiviral pathways, such as the interferon alpha/gamma responses and the defense response to virus, were strongly enriched, reflecting the induction of genes involved in viral sensing and restriction. At the same time, inflammatory and cytokine signaling pathways were also enriched (Fig. S5B), supporting a proinflammatory environment in peripheral blood. These results reinforce the hypothesis that ASFV infection triggers a robust but dysregulated immune response, characterized by strong transcriptional activation of antiviral and inflammatory pathways in peripheral blood cells that fails to achieve effective control of viral replication, potentially leading to immunopathology in the absence of vaccination.

For the vacc_t0 vs. ctrl_t0 condition, in which genes modulated by the effect of vaccination are revealed, a reduced but significant number of DEGs that are basally regulated by the vaccine prior to the challenge is evident (Fig. 6C). Downregulation of genes associated with a basal/resting profile (G0S2, AHRR, and PHLDA2), together with the upregulation of genes involved in immune surveillance (CD274, IL18RAP, and CCL4) or acute inflammation (PTX3), and those linked to proliferative readiness or DNA repair (RRM2, RAD54B, and EXO1), suggested that PBMCs from vaccinated animals were in a metabolically and immunologically alert state. Consistently, functional enrichment analysis revealed upregulation of pathways such as those involved in cell cycle and replication programs (HALLMARK_MYC_TARGETS_V1, HALLMARK_E2F_TARGETS, HALLMARK_G2M_CHECKPOINT, GOBP_DNA_TEMPLATED_DNA_REPLICATION) (Fig. 6F), further supporting the presence of proliferative and DNA replication signatures. This transcriptional profile was consistent with trained innate immunity and adaptive memory imprinting, as evidenced by the upregulation of CSF1, CCL4, HSPA4L, and MS4A7. These findings suggest an early modulatory effect of the vaccination with circulating PBMCs from vaccinated pigs at t0 transcriptionally primed for enhanced immune responsiveness at baseline.

Regarding the vacc_t4 vs. vacc_t0 condition, which evaluates the difference in gene expression between animals vaccinated prior to challenge (t0) and 4 days post-challenge (t4), no statistically significant DEGs were detected (padj <0.05). This transcriptional stability suggested the absence of a large-scale, dysregulated systemic response. GSEA analysis showed inhibition of cell cycle and replication (HALLMARK_E2F_TARGETS, HALLMARK_G2M_CHECKPOINT, and HALLMARK_MYC_TARGETS_V1), indicating that circulating PBMCs maintained the transcriptionally primed state established without engaging in cell cycle activation or proliferation (Fig. 6G). By contrast, upregulation of immune-related pathways, such as HALLMARK_TNFA_SIGNALING_VIA_NFKB and HALLMARK_INFLAMMATORY_RESPONSE, suggested that these cells remained in an alert, inflammatory poised state. Together, these results suggest that vaccination established a transcriptionally stable immune state that prevented unnecessary proliferation yet preserved readiness to respond to challenge.

To determine how vaccination altered the host response to ASFV infection, transcriptomic profiles of immunized and unimmunized animals following challenge (vacc_t4 vs. ctrl_t4) were compared, revealing a total of 468 DEGs (padj <0.05) (Fig. 6D). Of these, 388 genes (approximately 83%) were significantly repressed, while 80 genes (approximately 17%) were overexpressed in vaccinated animals. Compared to controls, vaccinated pigs showed broad downregulation of chemokine and inflammatory mediators (CXCL10, CCL2, and SIGLEC1), antiviral genes and ISGs (ISG15, RSAD2, MX1, and IRF7), innate activation markers (FCGR1A, HERC5/6, PLSCR1, and STAT1), and stress response genes (HSP70.2, HSPH1, DUSP1, and CASP3), effectively limiting pathological inflammation. The downregulation of the immune checkpoint CD274 likely indicated reduced need for immunosuppressive braking in the absence of clinical inflammation. On the other hand, genes encoding trafficking receptors for cytotoxic cells (CXCR3 and CXCR6) and markers of their activation and effector function (GZMK, KLRK1, KLRD1, and CRTAM) were upregulated, indicating an enhanced cytotoxic potential for cytotoxic cells to migrate to and eliminate infected targets, a precise immune response without systemic overactivation. GSEA showed strong inhibition of antiviral and interferon-related pathways, e.g., HALLMARK_INTERFERON_ALPHA/GAMMA_RESPONSE and GOBP_DEFENSE_RESPONSE_TO_VIRUS, indicating suppression of systemic antiviral and inflammatory signaling (Fig. 6H). T cell developmental programs (GOBP_T_CELL_LINEAGE_COMMITMENT and GOBP_V_D_J_RECOMBINATION) were also downregulated, suggesting that the immune response was mediated by pre-existing peripheral T cell populations, not by thymic output. In contrast, enrichment of HALLMARK_E2F_TARGETS and HALLMARK_G2M_CHECKPOINT suggested engagement of cell cycle–related programs, consistent with proliferative activity in a subset of antigen-activated T cells. These results support a controlled, alert immune state in vaccinated animals, with dampened systemic inflammation but preserved cytotoxic readiness.

### Vaccination does activate the immune response in a controlled manner

To explore the global expression patterns between the different experimental conditions showed above and to detect genes with either coordinated or divergent regulation, a cross-analysis of differential expression was performed by comparing the log2FC values of the genes in pairs of independent contrasts.

**Fig 7:**
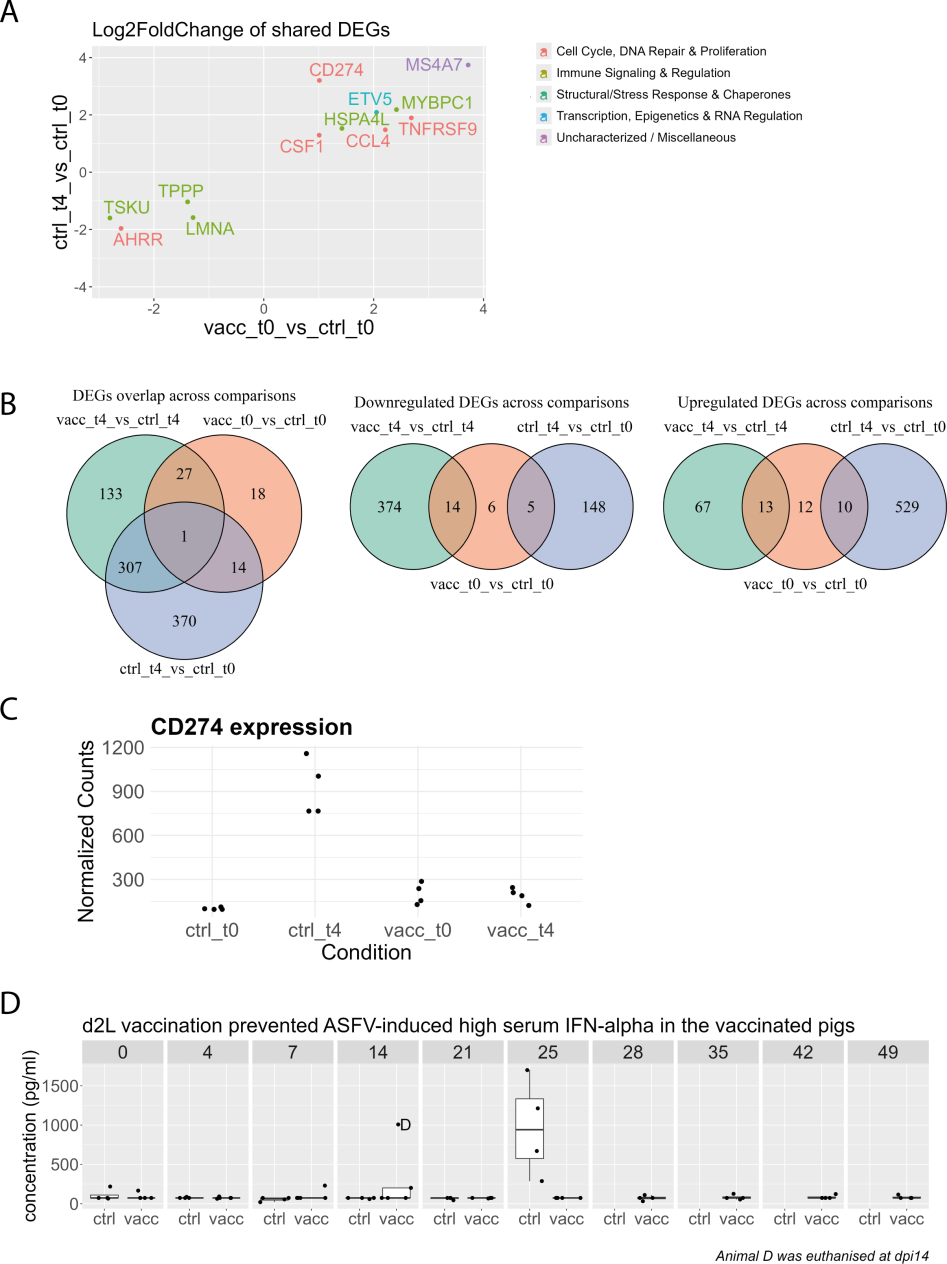
Differential gene expression analyses reveal that vaccination induces immune responses similar to ASFV infection with enhanced regulation. (**A**) Pairwise comparisons of log₂ fold change (log₂FC) values between vacc_t0_vs_ctrl_t0 vs. ctrl_t4_vs_ctrl_t0. Each dot represents a gene. (**B**) Venn diagram showing the overlap of differentially expressed genes (DEGs; FDR-adjusted *P* < 0.05) or exclusively up- or down-regulated expressed genes, as indicated. (**C**) Normalized expression counts of CD274 (PD-L1), highlighting condition-specific regulation. (**D**) IFN-α quantification in serum by ELISA in vaccinated and unvaccinated animals. The boxplot shows outlines (points outside whiskers), most extreme points within 1.5× interquartile range, 25th percentile (bottom of box), median (line inside box), and 75th percentile (top of box).

Direct comparison of vacc_t0_vs_ctrl_t0 and ctrl_t4_vs_ctrl_t0 revealed a shared set of downregulated (AHRR, TSKU, LMNA, and TPPP) and upregulated (CSF1, CCL4, TNFRSF9, CD274, ETV5, HSPA4L, MYBPC1, and MS4A7) DEGs, although with differing magnitudes relative to ctrl_t0 (Fig. 7A). In vaccinated pigs, these transcriptional changes were already established as part of a protective, primed state at baseline, whereas in naive pigs, they were only induced upon infection. This pattern suggests that vaccination may pre-activate certain immune pathways that are also induced by infection, but it does so in a more timely and regulated manner, thereby balancing the immune activation toward a controlled and protective response.

A Venn diagram illustrates the overlap and uniqueness of DEGs among key comparisons: disease progression without vaccination (ctrl_t4 vs. ctrl_t0), post-challenge status in vaccinated vs. unvaccinated animals (vacc_t4 vs. ctrl_t4), and baseline differences due to vaccination (vacc_t0 vs. ctrl_t0) (Fig. 7B). This latter comparison was used to exclude genes already modulated by immunization before challenge, allowing a more precise identification of infection-specific responses in vaccinated and unvaccinated animals. Notably, although many DEGs were shared between post-challenge vaccinated and unvaccinated animals (Fig. 7B, left panel), many of them exhibited opposite expression patterns, with no genes consistently up- or down-regulated in the same direction (Fig. 7B, central and right panels). This suggested that vaccination not only shapes which genes respond to infection but also reprograms the direction of their response, potentially mitigating the harmful effects of infection through a protective mechanism.

After comparing the three experimental conditions (ctrl_t4 vs. ctrl_t0, vacc_t4 vs. ctrl_t4, and vacc_t0 vs. ctrl_t0), CD274 was identified as the only differentially expressed gene present in all three comparisons (Fig. 7B). Mean normalized CD274 expression was low in controls (102 normalized counts) at baseline (ctrl_t0) but rose sharply to 924 (9-fold change) at ctrl_t4 (Fig. 7C), indicating strong induction in naive pigs, consistent with dysregulated inflammatory activation. In contrast, the vaccinated pigs (vacc_t0) showed moderately higher mean expression of 202 (2-fold change compared to ctrl_t0) that remained stable at 191 after challenge (vacc_t4), suggesting that vaccination pre-set CD274 expression and prevented infection-driven upregulation. The mean expression at ctrl_t4 was significantly higher than ctrl_t0 (*P* = 0.016), vacc_t0 (*P* = 0.015), and vacc_t4 (*P* = 0.016). Taken together, the results suggested that CD274 could be considered a marker of pathological disease progression and immune dysregulation and that vaccination counteracts an exacerbated immune response caused by ASFV infection.

Moreover, detection of IFN-α by ELISA supported these observations. As shown in Fig. 7D, IFN-α was detected in the serum of some vaccinated animals at 14 dpv, with particularly high levels in the animal that succumbed (pig D), establishing a link between pathogenicity and high levels of IFN-α. Consistent with this, high levels of IFN-α were only detected in unvaccinated animals after challenge at 4 dpc. The transcriptional profile described above in unvaccinated animals at t4 matched with high levels of IFN-α. In clear contrast, no IFN-α was detected in vaccinated animals. Therefore, while IFN-α indicates systemic immune activation, in this context, it is a specific marker of pathological inflammation and disease severity rather than protection.

In conclusion, transcriptomic studies suggested that Arm-ΔMGF110-2L modulated basal gene expression, which was sufficient to maintain stable transcriptional profiles in the vaccinated pigs upon challenge with virulent ASFV, thereby preventing virus-driven immune pathology. The restrained, basal-level CD274 expression in the vaccinated pigs and absence of serum IFN-α after challenge, in contrast to the strong upregulation observed in controls, likely contributed to the protective effect.

## DISCUSSION

ASFV is the causative agent of ASF, currently the biggest concern for pig farming worldwide, and is responsible for the largest animal pandemic affecting domestic pigs and wild boars. It is currently present in more than 60 countries on four continents, with the largest outbreaks occurring in Southeast Asia and Europe (13). Despite regionalization and other prevention measures, the disease has continued to spread since the first outbreak in the Caucasus in 2007, moving across Europe from east to west and currently affecting countries such as Germany and Italy (5, 6), and very recently Spain (7). The first outbreak in China in 2018 led to the disease spreading throughout the country and soon to neighboring countries in Southeast Asia, including Vietnam, South Korea, the Philippines, and, more recently, India and Indonesia (8–11).

There is still no safe and effective global vaccine, as due to the virus particularities, the most realistic vaccine option remains LAVs. This strategy involves deleting one or more genes involved in ASFV virulence from a virulent or attenuated strain to generate a fully attenuated prototype, yet inducing high levels of protection against a virulent challenge. In this regard, numerous prototypes that appear to be fully attenuated have been generated, providing protection up to 100% against virulent challenges (23–29, 34, 39). However, doubts have been raised about the safety of some of these prototypes after back-passage safety tests, where increased vaccine virus titers have been observed throughout passages in animals (33, 34). Another issue with the current vaccines is the lack of Differentiating Infected from Vaccinated Animals (DIVA) compatibility.

The search for new tools to protect against ASF, as well as the acquisition of fundamental knowledge about the molecular mechanisms of ASFV virulence, makes identifying ASFV genes involved in virulence one of the main topics of ASFV research. Previously, a relationship had been established between control over type I IFN production and ASFV virulence *in vivo* (40), particularly from genes belonging to MGF360 and MGF505 (40, 41). While not excluding other mechanisms of host response control, such as the NF-κB pathway (42, 43), TLRs (44), or the inflammasome (45, 46), it appears that the ASFV possesses numerous genes intended to control the type I IFN response. Recently, our group identified the cGAS/STING pathway as one of the main host defense factors targeted by ASFV virulent strains to control type I IFN production (19). From there, numerous ASFV proteins that control type I IFN production have been identified as virulence factors whose deletion causes attenuation of virulent strains (19, 20), and in this regard, we performed *in silico* screenings to identify new genes, such as MGF110-2L, which are absent or modified in attenuated strains. Not only that, but we found this gene absent in our previous recombinant Arm-ΔPolX-ΔMGFs mutant (36), most likely after successive passages *in vitro*. The MGF110-2L gene belongs to the MGF110 gene family, a set of genes that were identified early on near the 5′ end of the genome. These genes were probably generated by duplication and have a series of common characteristics. These include their early expression, a highly conserved cysteine-rich central domain, and a hydrophobic N-terminal domain. This suggests that they could pass through the secretory system (47). Proteins in this family have potential signal peptides (48), and this has been confirmed by MS for the MGF110-1L, 2L, 3L, and 4L genes (49). Ectopic assays indicated that MGF110-2L is capable of counteracting IFN-β production, a finding that was confirmed during infection with the knock-out virus (Arm-ΔMGF110-2L).

It looks like a large number of ASFV proteins act on cGAS/STING pathway, activated by sensing dsDNA in the cytoplasm of the cell (19, 37, 50, 51). Since ASFV is a dsDNA virus, it has evolved multiple proteins capable of counteracting the activation of this pathway at different levels. However, interestingly, MGF110-2L does not block the activation of this pathway, as neither does I329L, which antagonizes signaling through TLRs (44), or A238L, an IkB homolog inhibiting NF-kB signaling (42, 43). Other examples would be I215L, which controls type I IFN production via the NF-kB/AP-1 pathway (52), F317L, which stabilizes the IkB inhibitor and blocks NF-kB activity (53), and MGF360-12L, which inhibits type I IFN production by preventing NF-kB nuclear translocation (54). While many of these studies have been carried out using ectopic expression of these proteins, a direct relationship between these proteins and ASFV virulence has been demonstrated in some cases, as shown for A238L or MGF360-12L, depending on the genotype and other genes (28, 54, 55).

Additionally, there are other examples of ASFV proteins that are involved in controlling the innate immune response via pathways other than the cGAS-STING pathway, such as the inflammasome pathway. For example, B318L and H204R have been shown to inhibit both the NF-κB pathway and inflammasome activation by interacting with NLRP3 (45, 46). The deletion of the latter leads to attenuation of virulent genotype II strains (46), while the S273R protease inhibits the final execution of the inflammasome by preventing pyroptosis through the specific proteolysis of GSDMD (56). However, other proteins, such as I117L, have been described as activators of both the NF-κB pathway and the inflammasome. Specifically, I117L associates with NLRP3 and ASC, promoting ASC oligomerization (57).

Numerous studies have demonstrated the correlation between the absence of MGF gene clusters and ASFV virulence. First, the absence of several genes from the MGF360 and MGF505 families resulted in the attenuation of genotype I and genotype II strains (23, 41). Attenuation of large MGF gene clusters, along with others located at the 5′ end of the genome (and sometimes at the 3′ end), has typically resulted in the total or partial attenuation or adaptation of virulent strains (21, 36, 58, 59). However, the individual contribution of each of these genes has not been as thoroughly studied. In some cases, deleting one of these genes (37) or two of them does lead to the complete (60, 61) or partial (62) attenuation of virulent isolates. Conversely, the deletion of several others does not appear to affect virulence (63, 64). The function of these genes has primarily been investigated *in vitro* in relation to type I IFN control mechanisms or other host mechanisms. However, the impact of these genes on ASFV virulence *in vivo* remains to be investigated (see (20) for further details).

Regarding the MGF110 family, the function of some of these genes has been studied in the context of ASFV virulence. The absence of approximately 14 kb, including all MGF110 genes, led to partial attenuation of the natural (Estonia 2014) isolate (59). However, the individual deletion of any of these genes produces different results. For instance, deleting MGF110-1L or MGF110-5L-6L had no effect on ASFV virulence (63, 64), while deleting MGF110-9L or MGF110-11L produced partial attenuation of the deletion mutants (65, 66), as was also seen with MGF110-2L. It is worth asking why the deletion of all MGF110 genes, along with several others, produces similar or lesser attenuation in Estonia 2014 than the individual deletion of any of these genes. However, genetic differences found in Estonia 2014, such as gene duplication at the 3′ end (59), could explain this apparent contradiction.

We show here that MGF110-2L is directly involved in ASFV virulence since deletion of this gene causes partial attenuation of the Arm/07/CBM/c2 virulent strain, with four out of five immunized animals surviving throughout the experiment. However, while the absence of the MGF110-2L gene is the major genetic change in the Arm-ΔMGF110-2L recombinant virus, other minor genetic changes detected in this recombinant, such as the SNP that causes the Y307H mutation in the EP424R gene, may also contribute to the observed attenuation. Moreover, since one of the animals was euthanized during immunization and the rest of the vaccinated animals showed certain clinical signs, it cannot be ruled out that a higher vaccine dose could have been fatal for some of them. On the other hand, surviving pigs were challenged with parental virulent virus, showing no signs of pathology after 28 dpc, whereas the control animals succumbed between 6 and 7 dpc.

To understand the immunoprotective mechanisms triggered by this LAV prototype, we performed a transcriptomic analysis of PBMCs using RNA sequencing. PCA of the RNA-seq data revealed that the samples from unvaccinated animals at 4 dpc were distinct from the other samples (unvaccinated t = 0, vaccinated t = 0 and t = 4), suggesting significant transcriptional differences in this group. This is consistent with other studies indicating a significant transcriptional difference in animals inoculated with virulent ASFV strains, resulting in a cytokine storm (15, 16). Conversely, animals vaccinated at 4 dpc were grouped with control and vaccinated animals at 0 dpc, suggesting the minimal transcriptional disturbances following the challenge. The latter suggests that vaccination causes minimal transcriptional changes in vaccinated animals before and after challenge.

Our results show a significant number of DEGs observed in unvaccinated animals after challenge (ctrl_t4 vs. ctrl_t0), the vast majority of which are upregulated. These include type I IFN response, chemokines, and antiviral genes. GSEA indicates significant activation of antiviral pathways and the IFN response, as well as cytokine signaling and inflammatory pathways. These findings support the hypothesis that ASFV infection leads to an unregulated immune and inflammatory response, which could contribute to ASF pathology, and are consistent with several other studies describing virulent ASFV strains as responsible for a cytokine storm and ASF pathology from 3 to 5 dpc (15, 16, 67, 68). At the transcriptomic level, significant changes in the number of DEGs have also been observed elsewhere, with increased expression of genes involved in the cytokine response, innate immune response, and inflammation in organs such as the spleen, submandibular lymph node (LN), mesenteric LN, inguinal LN, tonsils, lungs, liver, kidneys, and heart at 3 dpc (69). These changes gradually increased in PBMCs from 2 to 8 dpc (16) and in whole blood from 5 dpc onward (67), coinciding with the detection of cytokines in serum (15, 16). These results are consistent with our observations at 4 dpc in unvaccinated control animals, reinforcing the idea that cytokine storm production and immune/inflammatory system regulation contribute to ASF pathology. Other scRNA-seq studies have also observed an increase in the expression of proinflammatory and innate immune response genes in spleen cells at 5 dpc. However, it should be noted that the cells in which this overexpression occurs are uninfected bystander cells (68), which is a finding that cannot be corroborated with the current RNA-seq data. While most DEGs are upregulated, we also observed the downregulation of genes involved in cellular metabolism and repair, according to studies that have shown the early (1 dpc) downregulation of genes involved in peptide translation and metabolism (67).

Regarding the vaccine footprint (vacc_t0 vs. ctrl_t0 animals), an active metabolic and immune state is observed in the primed PBMCs, in contrast to the inhibition of pathways involved in replication and cell cycle, whereas pathways involved in the inflammatory response were activated. The latter has also been observed to be upregulated in the organs of animals infected with virulent genotype II ASFV strains at the early stages of infection (69).

Surprisingly, no DEGs appear in vaccinated animals after challenge (vacc_t4 vs. vacc_t0). This suggests that vaccination with our prototype Arm-ΔMGF110-2L induces an immune state in circulating PBMCs that remains largely unaffected by challenge with virulent ASFV strains. Functional enrichment analysis revealed the downregulation of proliferation pathways and the activation of inflammatory and NF-κB pathways, which have previously been observed during infection with virulent strains (67, 69). Altogether, this suggests a more controlled inflammatory immune response in vaccinated animals.

Finally, when we compare the results observed at 4 dpc between vaccinated and unvaccinated animals (vacc_t4 vs. ctrl_t4), we observe a significant downregulation, 83% of total genes, mostly corresponding to inflammatory mediators, cytokines, markers of innate immune response activation, and stress response genes in vaccinated pigs. This would demonstrate that vaccination modulate the dysregulated immune and inflammatory response, causing the ASF-associated pathology during virulent infection. Since other studies indicate a change in tropism from macrophages to monocytes in spleen cells at long post-infection times (68), it would be interesting to study whether this or other changes in cell composition occur in PBMCs at these long time points. It would also be interesting to study what happens in vaccinated animals, since this change in tropism may contribute to the virulence and protective mechanisms against ASFV. However, vaccination with other LAV models, such as Ba71-ΔCD2v, has resulted in the upregulation of ISGs, TNFα, and inflammatory mediators that have been reported in PBMCs from vaccinated animals compared to unvaccinated animals (70). Importantly, it should be noted that among other methodological differences (oro-nasal immunization and contact challenge), RNA-seq analysis of the Ba71V- ΔCD2v LAV model was performed on PBMCs after *in vitro* stimulation with ASFV. These experimental conditions appear to more closely mimic the conditions we observed when comparing vaccinated and unvaccinated animals prior to challenge (vacc_t0 vs. ctrl_t0) than the distinct responses to the challenge observed in vaccinated versus unvaccinated animals (vacc_t4 vs. ctrl_t4). It would be interesting to analyze whether LAV Ba71-ΔCD2v behaves similarly to LAV Arm-ΔMGF110-2L under the same experimental conditions.

A direct comparison of DEGs in vaccinated and unvaccinated animals after the challenge with respect to the control showed the same set of DEGs, but with lower magnitude, in vaccinated animals. Furthermore, when we use a Venn diagram to compare the DEGs between the different conditions, we find a series of common DEGs comparing vaccinated vs. unvaccinated animals after challenge. However, the expression pattern of these genes is inverted between the two conditions, with the genes being up- or down-regulated in opposite directions. These data reinforce the idea that the vaccine modifies the transcriptomic landscape in a manner similar to the virulent challenge, albeit in a more controlled way.

The Venn diagram comparison of DEGs also identifies a single gene that varies between the three studied conditions: CD274. This gene is overexpressed in vaccinated animals both before and after the virulent challenge, the highest expression appearing in unvaccinated animals after challenge. Therefore, depending on its expression levels, it could be considered either a marker of disease progression or a marker of protection. CD274 encodes the PD-L1 protein, which binds to the PD-1 receptor to suppress the immune response of T cells. This interaction transmits an inhibitory signal that slows the activation, proliferation, and effector function of T cells, maintaining peripheral tolerance and preventing autoimmunity (71). However, sustained expression of this gene in pathological conditions causes an inefficient T cell response and generates a sustained but inefficient inflammatory response (72). Its role in both immune homeostasis and the dysregulation of inflammation, depending on its expression levels, could be consistent with the protective immune response induced by LAV and the pathogenesis associated with ASF. Other *in vivo* transcriptomic studies have identified host genes that could play a significant role in ASFV-associated pathogenesis. These include DUSP1, a restriction factor for ASFV infection that appears to be downregulated in infected macrophages (68), and genes of the Netrin axis (NTN4, DCC, and NEO1), which also appear to be downregulated during ASFV infection (73). Studying the mechanisms involving all these proteins will improve our understanding of ASFV-induced pathogenesis and contribute to the design of new vaccines.

Another marker of pathogenesis is the production of IFN-α, which has been described in this and other studies (74). In fact, IFN-α is practically undetectable in the serum of vaccinated animals, except in an animal that succumbed on 14 dpv. Furthermore, IFN-α is also undetectable in vaccinated animals after a virulent challenge, whereas a significant increase is observed in unvaccinated animals at 4 dpc, in line with the cytokine storm associated with ASF pathogenesis described elsewhere (15, 16). It may seem contradictory that the absence of MGF110-2L, a gene identified *in silico* and *in vitro* as a suppressor of type I IFN production, nevertheless prevents the overproduction of IFN-α *in vivo* mediated by the virulent ASFV strain Arm/07/CBM/c2. In fact, the absence of MGF110-2L in LAV Arm-ΔMGF110-2L induces significantly higher levels of type I IFN production than Arm/07/CBM/c2 in PAMs *in vitro*—the opposite of what occurs *in vivo*. This had already been observed in other ASFV mutants lacking genes involved in type I IFN control (belonging to MGF360 and MGF505). The absence of these genes in a deletion mutant produced a decrease in circulating IFN-α in domestic pigs compared to inoculation with the virulent parental strain (75). The most plausible explanation comes from scRNA-seq studies in *in vivo* ASFV models, which have determined that populations of macrophages, monocytes, and other immune cells responsible for producing type I IFN and proinflammatory cytokines are not infected cells, but bystanders (68). Other *in vitro* studies have recently corroborated this, demonstrating that plasmacytoid dendritic cells (pDCs) are pivotal in the production of IFN-α following the detection of infected cells (76). Therefore, cells infected with the Arm-ΔMGF110-2L prototype may produce a signal that prevents bystander cells (from the bloodstream and/or other immune tissues) from producing excessive cytokines and inflammatory mediators, although the molecular mechanisms remain to be elucidated.

In conclusion, these findings suggest that Arm-ΔMGF110-2L is a promising candidate for developing new protective tools based on LAV vaccines. In addition to identifying a new virulence gene and generating a new vaccine prototype, this study has advanced our understanding of the protective mechanisms triggered by the prototype. Further studies involving other prototypes will improve our understanding of the mechanisms underlying protection against ASFV and contribute to the development of new tools for controlling this devastating disease.

## MATERIALS AND METHODS

### Cell lines and viruses

*Cercopithecus aethiops* kidney cells (COS-1 cells), human embryonic kidney cells (HEK 293T cells), and HeLa cells were obtained from the American Type Culture Collection. They were both grown in Dulbecco’s modified Eagle’s medium (DMEM) supplemented with 2 mM of L-glutamine, 100 U/mL of gentamicin, and non-essential amino acids, plus 5% fetal bovine serum (FBS) (SIGMA Life Science). PAMs were provided by Agropardal (Cuenca, Spain) and extracted from bronchoalveolar lavage as previously described (77). They were cultured in DMEM supplemented with 10% porcine serum.

Viruses used were the ASFV viral strain Arm/07/CBM/c2 (LR812933.1) (78), NH/P68 (NC_044943.1) (18), the recombinant virus generated in this work, Arm-ΔMGF110-2L, and the recombinant virus VSV-GFP kindly gifted by Bruno Hernáez (CBM, Madrid, Spain). Both Arm/07/CBM/c2 and NH/P68 were produced in PAMs, while Arm-ΔMGF110-2L was produced in COS-1. Briefly, subconfluent PAM or COS-1 cells were cultivated in p150 plates and infected with ASFV at an MOI of 0.2 PFU/cell in DMEM-10% porcine serum. At 96 hpi, the cells were recovered and centrifuged at 3,000 rpm for 15 min. The cell pellet was discarded. The supernatant containing the viruses was clarified at 14,000 rpm for 6 h at 4°C, resuspended in medium, and stored at −80°C.

### Sequence comparison of ASFV genomes representing strains of different virulence

In order to identify viral genes potentially involved in ASFV virulence, eight different ASFV genomes from genotype I and II were obtained from GenBank. In particular, it encompassed three attenuated (NH/P68, OURT 88/3, and Estonia 2014), four virulent (L60, E75, Georgia 2007/1, and China/2018/AnhuiXCGQ), and one adapted (Ba71V) strain. An *in silico* analysis was performed using BLASTp for the alignment of the protein sequences from all these strains with each other. The output alignment was then processed using the Markov Cluster Algorithm (MCL) (79), with an inflation value of 4, which clustered the proteins according to their similarity into 165 different clusters comparing eight strains, which were manually analyzed.

### Vectors and cloning

For the luciferase assay, pIFN-β-luc, pRLTK, and pTBK1-Flag were kindly gifted by Adolfo García-Sastre (Icahn School of Medicine at Mount Sinai, NY, USA).

For viral gene expression, ASFV genes were amplified by PCR from Arm/07/CBM/c2 (LR812933.1) and cloned into pcDNA-3.1(+)-myc-HisB (Invitrogen) by using the InFusion Technology (Clonetech). Vector was linearized and genes amplified by using Phusion High-Fidelity PCR Master Mix (Thermo Fisher Scientific, MA, USA) using the oligos (A) shown in Table S3.

For recombinant virus generation by CRISPR/Cas9, we generated the vectors pFL-MGF110-2L, pFLΔMGF110-2L-p72-GFP, pSpCas9(BB)ΔNLS-2APuro_MGF110-2L-gRNA-0, and 1 by using the oligos (B) (Table S3). pFL and pFLΔ vectors were generated by InFusion Technology (Takara), and pSpCas9(BB)ΔNLS-2A-Puro were generated as previously described, including gRNA sequence 5′-GGCCAGCTAGCAGCAAGCCGAGG-3′ (gRNA-0) or 5′-GACAGGAGCATAGCTATCCCATGG-3′ (gRNA-2), respectively.

### Transfection

For transfection, Metafectene Pro transfection reagent (Biontex, München, Germany) or FuGene HD transfection reagent (Promega, Madison, WI, USA) was employed, following the manufacturer’s instructions. Briefly, 2 µg DNA/10^6^ cells were incubated with either Metafectene or FuGene at a 1:3 ratio in OptiMem (Gibco, Thermo Fisher Scientific, MA, USA) reduced serum medium for 15–20 min at RT and then added to cells and further incubated for 6 h. Then, the transfection mixture was replaced by DMEM 5% FBS, and cells were incubated at 37°C, 5% CO_2_ for subsequent experiments.

### Luciferase assay

HEK 293T cells were seeded on M24 plates (1.5 × 10^5^ cells/well) and co-transfected with the plasmids pIFN-β-luc (50 ng/well) and Renilla luciferase reporter construct pRLTK (25 ng/well) together with TBK1-Flag (50 ng/well) and either viral genes cloned in pcDNA-3.1(+)-myc-HisB (1,000 ng/well) plasmids or empty vector. Transfection was performed using FuGene HD as explained above. At 24 hpt, cells were collected and processed as indicated by the manufacturer of the Luc-PairTM Duo-Luciferase HS kit (GeneCopoeia). Finally, luciferase activity was measured using the FLUOstar OPTIMA reader (BMG LabTech, Ortenberg, Germany).

### Western blot and antibodies

At the indicated condition, the cells were washed with phosphate buffered saline (PBS) and lysed by RIPA lysis buffer (50 mM Tris-HCl pH = 7.4, 150 mM NaCl, 1% Triton, 0.5% sodium deoxycholate, 0.1% SDS). Samples were incubated at 4°C for 30 min. Then, they were sonicated and centrifuged for 8 min at 13,500 rpm at 4°C, and the pellet was discarded. Protein concentration was measured using the bicinchoninic acid (BCA) Protein Assay Kit (Pierce, Appleton, WI, USA), a spectrophotometric quantification method according to the manufacturer’s instructions.

Equal amounts of total protein from each sample were mixed with 5× Laemmli loading buffer (1 M Tris buffer pH = 6.8, 10% SDS, 4% β-mercaptoethanol, 20% glycerol, and 0.01% bromophenol blue, Solon, Ohio, USA) and incubated at 95°C for 5 min. Proteins were separated by electrophoresis on a 7%–20% polyacrylamide-SDS gel (SDS-PAGE) and transferred to Immobilon-P membranes (Millipore, Burlington, MA, USA). Membranes were blocked with Tris Saline buffer (TBS) with 5% skimmed milk powder for 1 h at RT before being incubated with the indicated primary antibodies diluted in TBS with 1% skimmed milk powder at 4°C overnight, and subsequently with the corresponding peroxidase-coupled secondary antibody. Finally, the identification of specific protein bands was performed using the ECL Western Blotting Analysis System chemiluminescence method (Amersham Pharmacia, Peapack, NJ, USA).

Primary antibodies anti-Myc (clone 9B11) (2276), DYKDDDDK Tag antibody (#2368), anti-pTBK1/NAK (Ser172) (D52C2), anti-TBK-1 E8I3G (38066), anti-pSTING Ser366 (85753), and anti-p-IRF3 Ser396 (4947) were purchased from Cell Signaling Technology (Danvers, MS, USA); anti-actin (C-4) (Sc-47778) was purchased from Santa Cruz (Santa Cruz, CA, USA); anti-STING (19851-1-AP) was purchased from Proteintech (Rosemont, IL, USA); and anti-p32 (S-1D8) was previously generated. Secondary antibodies anti-rabbit (NA934VS) and anti-mouse (NXA931V) coupled to peroxidase antibodies were purchased from Amersham Biosciences (Buckinghamshire, UK).

### Generation of Arm-ΔMGF110-2L by CRISPR/Cas9 technology

COS-1 cells were co-transfected with 2 µg of pFLΔMGF110-2L-p72-GFP and 2 µg of pSpCas9(BB)ΔNLS-2APuro_MGF110-2L-gRNA-0 or 1 with FuGene HD (Promega, Madison, WI, USA). At 24 hpt, 10 µg/mL of puromycin (SIGMA, Saint Louis, MO, USA) was added for selection of transfected cells; 48 h after the addition of puromycin, cells were infected with Arm/07/CBM/c2, collected 72 h post-infection (hpi), and stored at −80°C.

Collected samples were used to infect COS-1 cells. After 2 h of absorption, the inoculum was removed, and the cells were covered with carboxymethylcellulose (Sigma, Saint Louis, MO, USA) in DMEM 2% fetal bovine serum medium. Viral plaques were identified with a fluorescent microscope 3–7 dpi, and they were collected in 30 µL of DMEM and stored at −80°C. These viral plaques were freeze-thaw three times and used to infect COS-1 cells, repeating this procedure until the recombinant virus was isolated from its parental virus. To detect the presence of the recombinant and parental viruses, the digested isolated plaque was used as a DNA template. For that, 10 μL of the isolated plaque was digested with proteinase K (Sigma, Saint Louis, MO, USA) in 1.5 mM MgCl2, 50 mM KCl, 0.45% Tween 20, 0.45% NP40, and 10 mM Tris-HCl pH 8.3 buffer, incubated for 30 min at 45°C, and for 15 min at 95°C. The isolation was checked by PCR, with specific oligos shown in Table S3.

### Viral DNA extraction for NGS analysis

Recombinant Arm-ΔMGF110-2L was amplified on COS-1 cells (1.6 × 10^7^), and at 3 dpi, viral DNA from extracellular particles was extracted as previously indicated (78). Briefly, supernatant was centrifuged at 6,000 rpm overnight at 4 °C. Pellet obtained was resuspended in cold, filtered 10 mM Tris-HCl (pH = 8.8), and then treated with DNAase I (Sigma) 0.25 U/mL, Nuclease S7 (Sigma) 0.25 U/mL, and RNAase A (Promega) 20 μg/mL in nuclease buffer (0.8 M Tris-HCl, pH = 7.5, 0.2 M NaCl, 20 mM CaCl2, 120 mM MgCl2) for 2 h at 37 °C. Next, 12 mM EDTA pH = 8, and 2 mM EGTA were added and incubated for 10 min at 75°C. Then, the sample was treated with proteinase K (Sigma) at 200 μg/mL in 0.5% SDS for 1 h at 45 °C. For viral DNA precipitation, phenol:chloroform:isoamyl alcohol (25:24:1) was added in 1:1 proportion, and the sample was centrifuged (10,000 rpm, 3 min at RT). The aqueous fraction was transferred to another Eppendorf tube, and 0.1 volumes of 3 M acetic acid (pH = 5.2), 2 volumes of cold 100% ethanol, and 1 μL of LPA (Sigma) were added. The mix was incubated at −80°C o/n. Then, the sample was centrifuged (13,500 rpm, 30 min at 4°C), and the supernatant was discarded. Finally, the pellet was washed with cold 70% ethanol twice, air dried, and finally resuspended in 10 mM Tris (pH = 8.8).

### Illumina sequencing and data analysis

Recombinant Arm-ΔMGF110-2L virus DNA was sequenced with Illumina technology at MicrobesNG (Birmingham, UK) using MiSeq. Reads are analyzed as described elsewhere (56). Reads were trimmed with trimmomatic software (v0.39), and quality analysis was performed with FastQC software (v0.11.8). Reads were aligned to the Arm/07/CBM/c2 parental genome (LR812933.1) using Bowtie2 (v2.3.5.1) and samtools (v1.1), and indexed BAM files were visualized with IGV software (Integrative Genomics Viewer, v2.8.6) using the Arm/07/CBM/c2 genome as reference. For variant analysis, GATK software (v4.1.2) was used with standard parameters, and variants were mapped to Arm/07/CBM/c2 ORFs using SNPEff software (v4.3t). A coverage plot of Arm-ΔMGF110-2L reads against Arm/07/CBM/c2 parental genome (LR812933.1) was generated using bedtools (v2.27.1) for coverage values calculation and RStudio software (v2022.02.3 + 492) for histogram generation using the plot function.

### Viral genome assembly

Assembly of viral reads, showing over 80% of alignment over the parental Arm/07/CBM/c2 genome, was performed using Unicycler (v.0.4.8) (80) with standard parameters and Illumina-only mode. The quality of obtained contigs was assessed with Quast software and aligned against the parental Arm/07/CBM/c2 reference genome using nucleotide BLAST+ (v 2.5.0+) to select contigs corresponding to the ASFV genome.

Selected contigs were aligned against *in silico* Arm-ΔMGF110-2L and parental Arm/07/CBM/c2 assemblies using the LAST aligner to manually select and orient the matching contigs correctly. Illumina reads were aligned against the oriented contigs using Burrows-Wheeler aligner (BWA) (81), sorted by coordinate, and indexed with samtools. Ends were elongated using flye software and manual curation against previous assemblies, and elongated ends were verified against Illumina reads. The obtained genome coverage was analyzed using the GenomeCoverageBed command, showing a length of 193,174 base pairs and a mean coverage of 1,311.93 reads per base.

Finally, the *de novo*-assembled genome was annotated with a combination of Prokka (82) and Rapid Annotation Transfer Tool (RATT) (83) to avoid missing genes.

### Viral growth kinetics

Both single-cycle and multistep growth curves were performed. For the multistep growth curve, PAMs were seeded (1.67 × 10^6^ cells per well) and infected with either Arm/07/CBM/c2 or Arm-ΔMGF110-2L at MOI 0.5. Samples were collected at 0, 24, 48, 72, and 96 hpi. For the single-cycle growth curve, PAMs were seeded (8.33 × 10^5^ cells per well) and infected with either Arm/07/CBM/c2 or Arm-ΔMGF110-2L at MOI 5. Samples were collected at 0, 4, 16, 24, and 48 hpi.

After three freeze/thaw cycles, samples were titrated by Reed & Muench TCID50 assay in COS-1 cells. Briefly, serial dilutions of each sample were used to infect COS-1 cells seeded in a p96 well plate (7,000 cells/well). At 72 hpi, cells were fixed with PBS 4% paraformaldehyde (PFA). Samples were permeabilized with 0.2% Triton-X-100 and stained with viral primary antibody anti-p32 (S-5C1) (1:500) incubation, followed by anti-mouse Alexa Fluor 488 secondary antibody (A-21206) incubation, and infection was observed by green fluorescence in a fluorescence microscope. Biological duplicates were used. Data were statistically analyzed by ordinary two-way ANOVA (**P < 0.01; ***P < 0.001).

### RT-qPCR assays

For ectopic expression assays, HEK 293T cells were seeded (4 × 10^6^ cells/well) and co-transfected with pTBK-1-Flag and either empty vector (pcDNA-3.1-myc-HisB) or pcDNA-MGF110-2L-myc-HisB. For infection assays, PAMs were seeded (8 × 10^6^ cells/well) and mock-infected or infected with either Arm/07/CBM/c2, NH/P68, or Arm-ΔMGF110-2L at MOI 2 for either 4 or 16 h. Biological and technical triplicates were used.

RNA was extracted using the RNeasy kit (Qiagen, Venlo, The Netherlands), and total RNA was quantified with NanoDrop One (Thermo Scientific). Equivalent amounts of RNA were then retrotranscribed using the NZY first-strand cDNA kit (NZYTech), and RT-PCR was performed in technical triplicate with a cDNA amount of 12.5 ng/sample on the CFX384 Touch Real-Time PCR system (Bio-Rad Laboratories, USA) thermal cycler using SYBR Green (NZYTech). Expression levels of genes of interest were normalized against 18S ribosomal RNA (rRNA) expression, and these values were relativized against the mean of the values obtained in the mock or the empty vector samples. Oligos used to run the qPCR are displayed in Table S4.

### Interferon bioassay

The presence of biologically active type I IFN in ASFV infection supernatants was detected by analyzing its ability to inhibit VSV infection (38). PAM cells (8.3 × 10⁵ cells/well) were seeded and infected with Arm/07/CM/c2, NH/P68, or Arm-ΔMGF110-2L at an MOI = 0.5, or left uninfected (mock). At 72 hpi, the cell culture medium was collected, centrifuged for 5 min at 1,500 rpm at 4°C, and incubated with psoralen for 10 min at room temperature, followed by UV irradiation for 20 min on ice (or not inactivated).

To verify proper inactivation of the viruses, COS-1 cells were incubated with inactivated or non-inactivated viral supernatants from Arm-ΔMGF110-2L infections, as well as being infected with Arm-ΔMGF110-2L (MOI = 1) to serve as a control for infection. At 8 and 16 hpi, samples were collected, washed in PBS, and incubated with Ghost Dye Red 780 (TONBO Biosciences) (1 µg/mL) for 5 min at 37°C in the dark, to distinguish between live and dead cells. The cells were then fixed with 2% PFA for 30 min at 4°C, after which they were analyzed using a FACSCanto A flow cytometer (BD Biosciences) to determine the percentage of cells expressing GFP (488+), which is indicative of infection with Arm-ΔMGF110-2L. Biological replicates were used.

Finally, for the IFN-I bioassay, HeLa cells were seeded in P96 wells (5,000 cells per well). After 24 h, the cells were incubated with decreasing concentrations of recombinant human IFN-β (hIFN-β) as a control, as well as with inactivated supernatants from the indicated infections. Twenty-four hours after incubation, both hIFN-β and the supernatants were removed, and the cells were infected with VSV-GFP (MOI = 2). GFP intensity was measured 16 hpi using a ClarioSTAR plate reader (Isogen Life Sciences, the Netherlands) to calculate the percentage of infected cells. VSV-GFP, psoralen, and hIFN-β were kindly provided by Bruno Hernáez (Antonio Alcamí’s laboratory, CBM, Madrid, Spain). Technical replicates (*n* = 5) were used. Data were statistically analyzed by one-way ANOVA (*P < 0.05; **P < 0.01; ***P < 0.001; ****P < 0.0001).

### Animal experimental conditions

A total of nine pigs, aged 6 weeks, both sexes, were divided into two experimental groups—Group I (Δ2L *n* = 5, pigs A–E) and control group (*n* = 4, pigs F–I). The animals have been purchased in a commercial local pig farm, with a confirmed high level of health status—serologically free from Porcine reproductive and respiratory syndrome virus, Aujeszky’s disease (PRV). The animals were acclimatized for 6 days in BSL3 (Biosafety Level 3) animal facility in two independent units with permanent access to feed and water. After the acclimatization phase, the health status of all pigs was evaluated by veterinary examination and confirmed to be free of ASFV by using VIRTOTYPE Real-time PCR kit (Indical Bioscience GMBH, Leipzig, Germany).

Group I was inoculated IM with 10^3^ TCID50 of the Arm-ΔMGF110-2L virus at 0 day post-vaccination (dpv). At 21 dpv, both groups were challenged IM with Arm/07/CBM/c2 virus at the dose 10^1^ TCID50. Humane endpoint criteria were applied if animals exhibited signs of severe suffering, including severe dyspnea, frothy nasal discharge, or severe recumbency.

### Sample collection and assessment of clinical signs

Clinical signs were assessed on a daily basis beginning from 0 dpv to 49 dpv (28 dpc). Fever was defined as a body temperature above 40.0°C for at least 2 consecutive days.

To assess viral load and shedding, rectal and oral swabs were collected at 0, 4, 7, 14, and 21 dpv and then 4, 7, 14, 21, and 28 dpc. Collected swabs were placed into tubes containing 1 mL of PBS, vortexed, and incubated (10 min, RT). At the same time points, blood was collected to plastic tubes containing K2-EDTA. Blood for serum was collected to serum separator tubes and centrifuged (1,800 G, RT); sera were stored at −20°C until further analysis.

Complete necropsy was done on each animal as soon as possible after death or euthanasia. Tissue samples (i.e., the spleen, liver, kidneys, lungs, submandibular lymph nodes, tonsils, and bone marrow) were collected to 2 mL tubes. About 10% dilution in PBS (wt/vol) of each tissue was done by homogenization in TissueLyser (Qiagen, Germany).

### ASFV qPCR and PCR-based differentiation between viruses

A total of 200 µL of each sample (i.e., oral and rectal swabs, blood [1:10 vol/vol PBS], tissue homogenates) was used for DNA extraction. Manual column extraction was performed according to Qiagen DNA Mini Kit protocol (Qiagen, Germany) or magnetic bead–based extraction was carried out with the IndiMag 48s system, in accordance with the manufacturer’s instructions. Real-time PCR was conducted according to VIROTYPE (Qiagen, Germany) manufacturer’s manual using the Rotor-Gene Q thermocycler (Qiagen, Germany) or an AriaMx system (Agilent, USA). A standard curve presenting dependence between Ct and virus titer was prepared (Microsoft Excel, Windows) as described previously (84). Relevant Ct values obtained during the experiment were estimated and expressed as equivalent of virus titer (eqTCID50/mL).

Virus differentiation analysis was performed using viral DNA isolated from stored samples. Conventional PCR was carried out in a total volume of 25 µL using HS Red MyTaq Mix (Meridian Bioscience, Cincinnati, OH, USA) on a Biometra thermocycler (Analytik Jena, Germany). The cycling conditions were as follows: initial denaturation at 95°C for 5 min and 35 cycles of 95°C for 30 s, 58°C for 30 s, and 72°C for 90 s, followed by a final extension at 72°C for 5 min. The wild-type virus yielded a PCR product of 1056 bp, whereas the recombinant Arm-ΔMGF110-2L virus produced a 781 bp fragment. Primer sequences are listed in Table S3.

### ELISA

The serological status of the serum samples was determined by enzyme-linked immunosorbent assay (ELISA) with ID Screen African Swine Fever Indirect (IDVet Innovative Diagnostic, Grabels, France) according to the manufacturer’s instructions.

In addition, 68 serum samples were analyzed for IFN-α production during immunization and challenge using a Porcine IFN-α ELISA Kit (Thermo Fisher Scientific, Carlsbad, CA), according to the manufacturer’s instructions.

### RNA-seq library preparation and sequencing

Bulk RNA-seq study included 16 PBMC samples, four vaccinated and four control pigs at 21 dpv (t0) and 25 dpv (t4). PBMCs were isolated from whole blood collected in EDTA tubes. The isolation was performed by density gradient centrifugation using Histopaque-1077 (Sigma-Aldrich, St. Louis, MO, USA) according to the manufacturer’s instructions. This method generally yielded sufficient PBMC numbers (~2 × 10⁶ cells per sample based on prior experience), although exact cell counts were not determined in this study. After centrifugation, the PBMC layer was carefully collected from the plasma-histopaque interface and washed with PBS. The cells were then resuspended in a cryopreservation medium consisting of 10% dimethyl sulfoxide (DMSO; PAN Biotech, Aidenbach, Germany), 20% FBS (Gibco, Thermo Fisher Scientific, Waltham, MA, USA), and 70% RPMI 1640 (PAN Biotech, Aidenbach, Germany), and stored at −80°C until further use.

The cryopreserved PBMCs were thawed in a 37°C water bath, and cells were transferred to 40 mL RPMI 1640 medium, centrifuged at 400 × *g* for 10 min, and washed once with 50 mL medium. The cells were lysed with 1 mL TRIzol reagent (Thermo Fisher Scientific, Carlsbad, CA). Total RNA was extracted with a combination of TRIzol reagent and RNeasy Mini Kit (Qiagen, Hilden, Germany) and eluted in 40 µL RNase-free water following the manufacturer’s instructions. RNA concentration and quality were measured by Fragment Analyzer or TapeStation system (Agilent, Santa Clara, CA). Libraries were constructed using TruSeq stranded mRNA preparation kit with polyA selection (Illumina Inc.) and paired-end sequencing with 150-bp read length was performed on a NovaSeq X Plus system and XLEAP-SBS sequencing chemistry (Illumina Inc.). Quality analysis of reads was performed using FastQC (v0.11.8), and adaptor trimming was done by Trimmomatic (v0.39).

### Bioinformatic analysis of RNA-seq data

The reads were aligned against the *Sus scrofa* reference genome (Assembly Sscrofa11.1, GCF_000003025.6) by using the HISAT2 (v2.1.0) aligner. Counting of the reads that had been mapped to reference gene annotation was performed by command htseq-count within Python package HTSeq (v0.11.2) under “intersection-strict resolution” model. Differential expression analysis was performed using R package DESeq2 (v1.49.1). DEGs were defined with adjusted *P*-value (padj) < 0.05. Then, in order to group the DEGs by pathways, a GSEA was performed (85) using R package fgsea (v1.28.0) against hallmark gene sets and ontology gene sets at Molecular Signatures Database (https://www.gsea-msigdb.org/gsea/index.jsp). Data visualization was performed using R package tidyverse (v2.0.0), VennDiagram (v1.7.3), RStudio (v2023.09.1), and R (v4.3.2) on x86_64-w64-mingw32/x64 platform. GO with *P*-value < 0.05 were selected.

## Data Availability

RNA-seq raw data and Illumina and genome assembly data are available at the European Nucleotide Archive (ENA) under accession no. PRJEB80166.

## SUPPLEMENTAL MATERIAL

Supplemental material jvi.00636-26-s0001.pdf Tables S1 to S4; Fig. S1 to S5.

ASM does not own the copyrights to Supplemental Material that may be linked to, or accessed through, an article. The authors have granted ASM a non-exclusive, world-wide license to publish the Supplemental Material files. Please contact the corresponding author directly for reuse.
