# Supplementary material for: MGF110-2L deletion prevents IFN-I and inflammatory response, resulting in partial attenuation and protection against virulent ASFV
Source: J Virol. 2026 Jun 26;100(7):e00636-26. doi: 10.1128/jvi.00636-26 (PMC13386828; doi:10.1128/jvi.00636-26)
Supplement: Supplemental material — Tables S1 to S4; Fig. S1 to S5. [file jvi.00636-26-s0001.pdf]

Supplementary material:

Table S1. Different virulent, partially attenuated, attenuated and adapted African Swine Fever virus strains included in the analysis. Accession number used at the time of analysis is included.

Strain ID	Strain Name	Genotype	Attenuated	Genome length (bp)
NC_044941.1	L60	I	No	182,362
NC_044958.1	E75	I	No	181,185
KM262845.1	NH/P68	I	Yes	172,051
AM712240.1	OURT 88/3	I	Yes	171,719
NC_001659.2	BA71V	I	Adapted	170,101
NC_044959.1	Georgia 2007/1	II	No	189,344
NMK128995.1	China/2018/AnhuiXCGQ	II	No	189,393
LS478113.1	Estonia2014	II	Partially	182,446

Table S2. Mutations identified in the deletion mutant virus Arm-ΔMGF110-2L by variant calling analysis compared with Arm/07/CBM/c2. The position corresponds to the position in the whole genome of Arm/07/CBM/c2.

Position	Type	Location	Mutation	Description
14005	InDel	MGF110-14L	A/AC	Loss of 6 aa N'term
15444	InDel	Intergenic region	A/AC	-
72293	SNP	EP424R	T/C	Tyr307His

Table S3. Oligos designed for PCR. A: oligos for cloning vectors for ectopic expression of ASFV genes in pcDNA-3.1-myc. B: oligos for vectors used for recombinant virus generation by CRISPR/Cas9. C: oligos for detection of recombinant or parental virus.

Oligonucleotides	Sequence (5'→3')
Lin_pcDNA_3.1_myc-Fw[A]	GAACAAAAACTCATCTCAGAAGAGG
Lin_pcDNA_3.1_myc-Rv[A]	GAA CCG CGG GCC CTC TAG
MGF110-2L-pcDNA-Fw[A]	AGACCCAAGCTGGCTAGTTAATGAGTTTCTTTAGTTACCTCGGC
MGF110-2L-myc-Rv[A]	ATGAGTTTTTGTTCGAACCGAGCATTTAATAAATGAATAGCATCACACG
MGF110-4L-pcDNA-Fw[A]	AGACCCAAGCTGGCTAGTTAATGTTGGTAGTCTTCTTGGGAATTCT

MGF110-4L-myc-Rv[A]	ATGAGTTTTTGTTCGAACCGCAGATCTTTCTTTTCATAAATTTTCATTAACAATTGGT
MGF110-11L-pcDNA-Fw[A]	AGACCCAAGCTGGCTAGTTAATGAAACTACTTGCTCTTTTATGTATTTTGAT
MGF110-11L-myc-Rv[A]	ATGAGTTTTTGTTCGAACCGATGACTAAACCCTTTATACGCTGT
MGF110-13L-pcDNA-Fw[A]	AGACCCAAGCTGGCTAGTTAATGAAGGTTCTTCTGGGACTTTTG
MGF110-13L-myc-Rv[A]	ATGAGTTTTTGTTCGAACCGACTAAAATATGTGGGCAGAGAGC
MGF110-5L-6L-pcDNA-Fw[A]	AGACCCAAGCTGGCTAGTTAATGTTGGTGATCTTCTTGGGAATTC
MGF110-5L-6L-myc-Rv[A]	ATGAGTTTTTGTTCGAACCGACTATTATTTTCTTTCCACTCTCTCTTTAAGATTT
MGF110-2L-pFL-Fw[B]	CCAGATATACGCGTTATGGTTGTGTTGTGGGCAT
MGF110-2L-pFL-Rv[B]	TTTCCGCCTCAGAAGATCTGCCAGCTGTTCAGC
pFLMGF110-2L-lin-Fw[B]	TGTAGTGTTATATTACGAAAAAACATGTTAAAATTTGGA
pFLMGF110-2L-lin-Rv[B]	CCGGGTTATTAGAAAACTCGTTATTTTTTTTATCA
EGFP-MGF110-2L-Fw[B]	TTTCTAATAACCCGGGACATTGATTATTGACTAGTTATTAATAGTAATCAATTACGG
EGFP-MGF110-2L-Rv[B]	TAATATAACACTACACCATAGAGCCCACCGC
gRNA_0_MGF110-2L-Fw[B]	caccGGCCAGCTAGCAGCAAGCCGAGG
gRNA_0_MGF110-2L-Rv[B]	aaacCCTCGGCTTGCTGCTAGCTGGCC
gRNA_2_MGF110-2L-Fw[B]	caccGACAGGAGCATAGCTATCCCATGG
gRNA_2_MGF110-2L-Rv[B]	aaacCCATGGGATAGCTATGCTCCTGTC
Arm-ΔMGF110-2L-VER_WT-Fw[C]	GCAGATGTATTGTCCAACAATCATGT
Arm-ΔMGF110-2L-VER_WT-Rv[C]	CTGGGCTTGTCAAGATGGTC
Arm-ΔMGF110-2L-VER_Rec-Fw[C]	CACAACCCACAAGGTAAAATCCAT
5'_GFP-Rv[C]	GAACTTGTGGCCGTTTACGT

Table S4. Oligos designed for RT-qPCR.

Oligonucleotides	Sequence (5'→3')
IFNβ_Homo_sapiens-Fw	TGAGAACCTCCTGGCTAAT
IFNβ_Homo_sapiens-Rv	CCCTGGTGAATTCTTCTTTCT
MGF110-2L-Fw	TTCGACCGACAATCCCCTG
MGF110-2L-Rv	TTGACAAGCCCAGCAAAACC
MGF110-1L-Fw	ACTGCAGTGTTTTTCGTCACAA
MGF110-1L-Rv	GTATGACAGCCTCGTGCCAT
MGF110-3L-Fw	TCTTGTGAGGTTTCTCGCTGG
MGF110-3L-Rv	TTAACAATTGGTGGGGGCGG
18S_Homo_sapiens-Fw	GCAATTATTCCCCATGAACG
18S_Homo_sapiens-Rv	GGGACTTAATCAACGCAAGC

IFNβ_Sus_scrofa-Fw	GTGGAACTTGATGGGCAGAT
IFNβ_Sus_scrofa-Rv	TTCCTCCTCCATGATTTCCTC
ISG15_Sus_scrofa-Fw	GGTGCAAAGCTTCAGAGACC
ISG15_Sus_scrofa-Rv	GTCAGCCAGACCTCATAGGC
B602L-Fw	GGTTTCATCCATTCAATTAGGC
B602L-Rv	CGCTCTCTGTTGGCCTTAAA
18S_Sus_scrofa-Fw	GGCCCGAGGTTATCTAGAGTC
18S_Sus_scrofa-Rv	TCAAAACCAACCCGGTCA

Figure S1. Cloning and expression of ASFV genes potentially involved in virulence. HEK 293T were transfected with empty vector (pcDNA-3.1-myc) or viral genes cloned in a pcDNA-3.1-myc (2μg/10^6 cells). 24h post transfection cells were lysed in RIPA buffer supplemented with phosphatase and protease inhibitors. Expression of indicated proteins were analyzed using Western blot with anti-myc and anti-actin.

Figure S2. Deletion of the MGF110-2L gene does not affect the expression of the upstream or downstream genes, MGF110-1L and MGF110-3L, respectively. PAM cells were infected with Arm/07/CBM/c2 or Arm-ΔMGF110-2L at an MOI of 2 or were left uninfected (MOCK). At 4 hpi, RT-qPCR was used to analyze the expression of MGF110-2L, MGF110-1L, and MGF110-3L. The data were statistically analyzed using one-way ANOVA (ns, $p > 0.05$; **, $p < 0.01$).

Figure S3. Optimization of the VSV-GFP Bioassay for Detecting Type I IFN. (A) To examine the correlation between type I IFN levels in the medium and the percentage of HeLa cells infected with VSV-GFP, the cells were incubated with decreasing concentrations of human interferon beta (hIFN-β). After 24 hours, the hIFN-β was

removed and the cells were infected with VSV-GFP for an additional 16 hours. At this point, fluorescence intensity (488 nm) was measured using a ClarioSTAR plate reader (Isogen Life Sciences) to calculate the infection percentage. (B) To verify the inactivation of ASFV-infected cell culture supernatant (SN), the SN was used to infect COS-1 cells either with or without psoralen/UV inactivation, derived from Arm-ΔMGF110-2L infections in PAM (MOI = 2). At 8 and 16 hpi, the cells were harvested, stained with fixable viability dye (Ghost Dye Red 780, 1 µg/mL), fixed, and analyzed by flow cytometry (FACScan, BD Biosciences) to determine the percentage of cells expressing GFP (indicative of infection).

Figure S4: PCR detection of Arm/07/CBM/c2 (WT) or Arm-ΔMGF110-2L. Detection of DNA corresponding to Arm/07/CBM/c2 (WT) (samples 1-7) or Arm-ΔMGF110-2L (samples 8-14) by specific PCR in (A) samples from vaccinated animals at 25 dpv (4 dpc) or (B) in tonsil samples obtained during necropsy. M: molecular weight marker. Samples 1 and 8 correspond to pig #A; samples 2 and 9, to pig #B; samples 3 and 10, to pig #C; and samples 4 and 11, to pig #E. Samples 5 and 12 are the Arm/07/CBM/c2 (WT) control; samples 6 and 13 are the Arm-ΔMGF110-2L control; and samples 7 and 14 are the negative control.

Figure S5. Transcriptomic profiling of PBMCs using RNA-seq reveals condition-dependent clustering and immune-related pathway activation. (A) Principal Component Analysis (PCA) of global gene expression from PBMCs of all experimental groups. Axes represent PC1 and PC2. (B) Gene Set Enrichment Analysis (GSEA) of PBMCs from unvaccinated animals comparing ctrl_t4 vs ctrl_t0, showing significantly

enriched biological pathways (adjusted p-value < 0.05), ranked by Normalized Enrichment Score (NES).

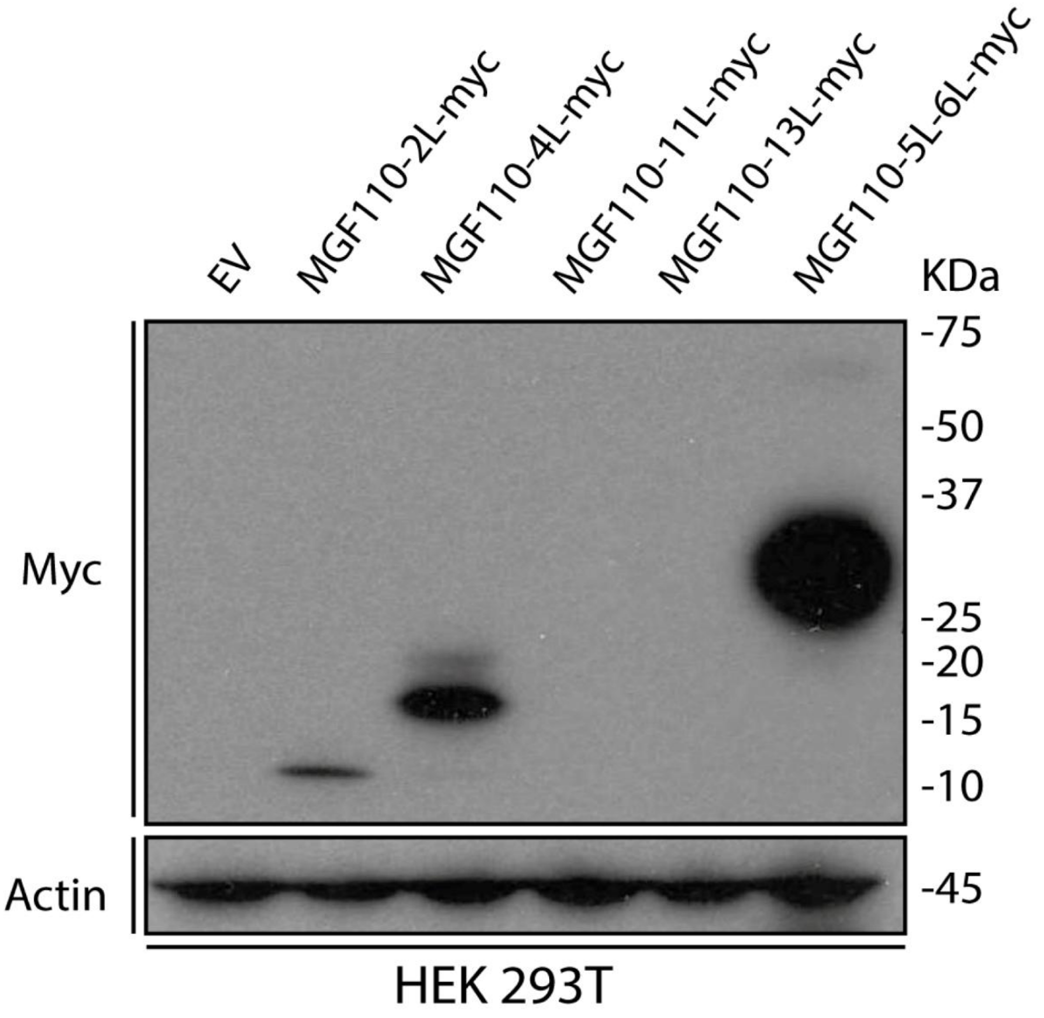

Figure S1. Cloning and expression of ASFV genes potentially involved in virulence. HEK 293T were transfected with empty vector (pcDNA-3.1-myc) or viral genes cloned in a pcDNA-3.1-myc (2µg/10^6 cells). 24h post transfection cells were lysed in RIPA buffer supplemented with phosphatase and protease inhibitors. Expression of indicated proteins were analyzed using Western blot with anti-myc and anti-actin.

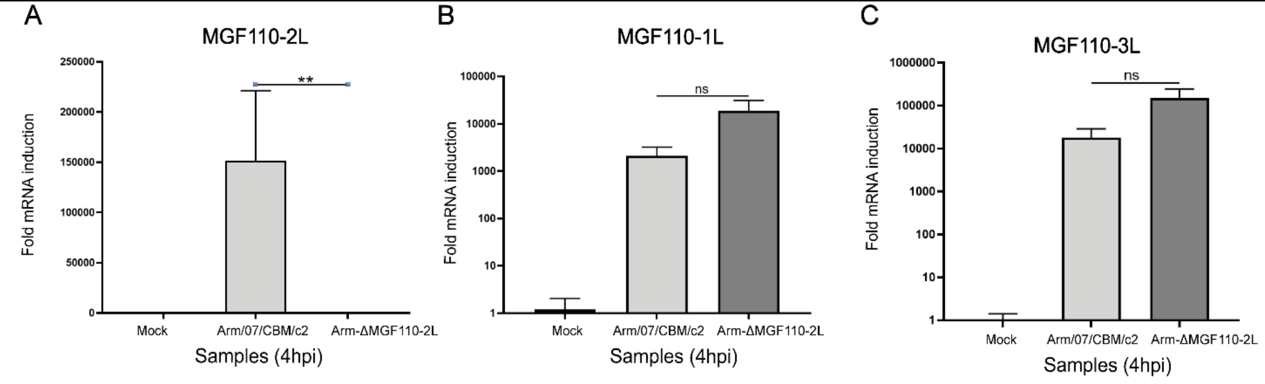

Figure S2. Deletion of the MGF110-2L gene does not affect the expression of the upstream or downstream genes, MGF110-1L and MGF110-3L, respectively. PAM cells were infected with Arm/07/CBM/c2 or Arm-ΔMGF110-2L at an MOI of 2 or were left uninfected (MOCK). At 4 hpi, RT-qPCR was used to analyze the expression of MGF110-2L, MGF110-1L, and MGF110-3L. The data were statistically analyzed using one-way ANOVA (ns, $p > 0.05$; **, $p < 0.01$).

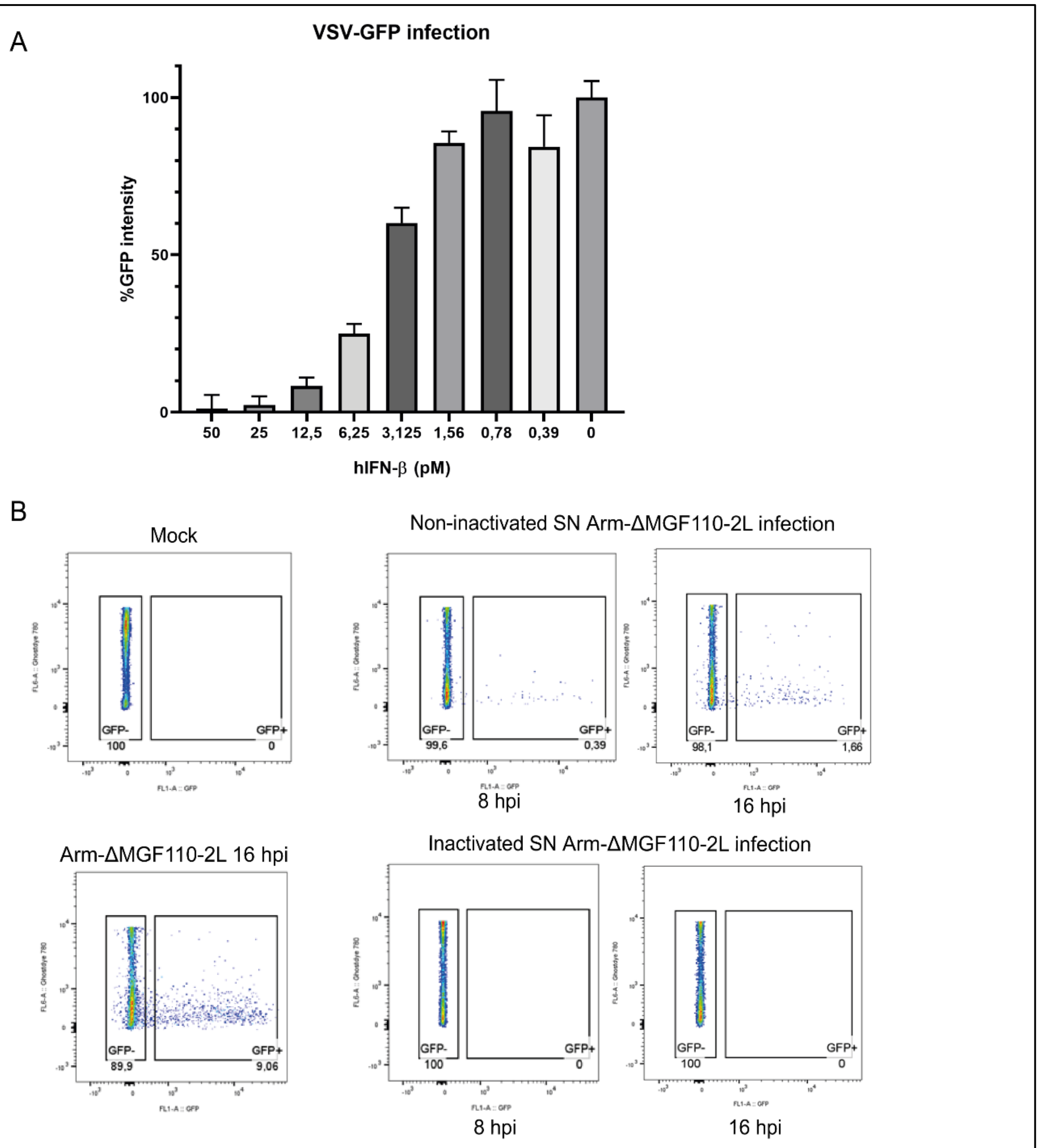

Figure S3. Optimization of the VSV-GFP Bioassay for Detecting Type I IFN. (A) To examine the correlation between type I IFN levels in the medium and the percentage of HeLa cells infected with VSV-GFP, the cells were incubated with decreasing concentrations of human interferon beta (hIFN-β). After 24 hours, the hIFN-β was removed and the cells were infected with VSV-GFP for an additional 16 hours. At this point, fluorescence intensity (488 nm) was measured using a ClarioSTAR plate reader (Isogen Life Sciences) to calculate the infection percentage. (B) To verify the inactivation of ASFV-infected cell culture supernatant (SN), the SN was used to infect COS-1 cells either with or without psoralen/UV inactivation, derived from Arm-ΔMGF110-2L infections in PAM (MOI = 2). At 8 and 16 hpi, the cells were harvested, stained with fixable viability dye (Ghost Dye Red 780, 1 µg/mL), fixed, and analyzed by flow cytometry (FACScan, BD Biosciences) to determine the percentage of cells expressing GFP (indicative of infection).

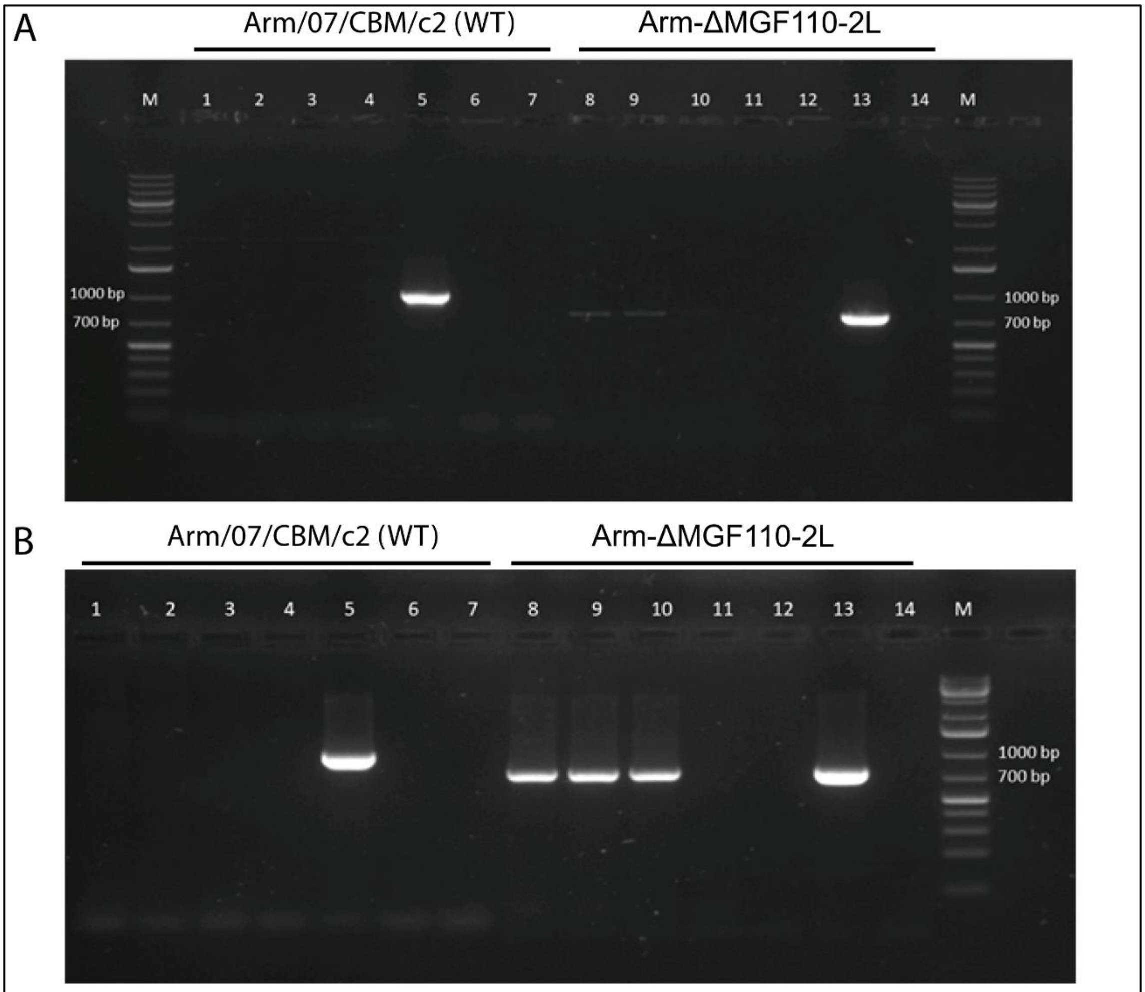

Figure S4: PCR detection of Arm/07/CBM/c2 (WT) or Arm-ΔMGF110-2L. Detection of DNA corresponding to Arm/07/CBM/c2 (WT) (samples 1-7) or Arm-ΔMGF110-2L (samples 8-14) by specific PCR in (A) samples from vaccinated animals at 25 dpv (4 dpc) or (B) in tonsil samples obtained during necropsy. M: molecular weight marker. Samples 1 and 8 correspond to pig #A; samples 2 and 9, to pig #B; samples 3 and 10, to pig #C; and samples 4 and 11, to pig #E. Samples 5 and 12 are the Arm/07/CBM/c2 (WT) control; samples 6 and 13 are the Arm-ΔMGF110-2L control; and samples 7 and 14 are the negative

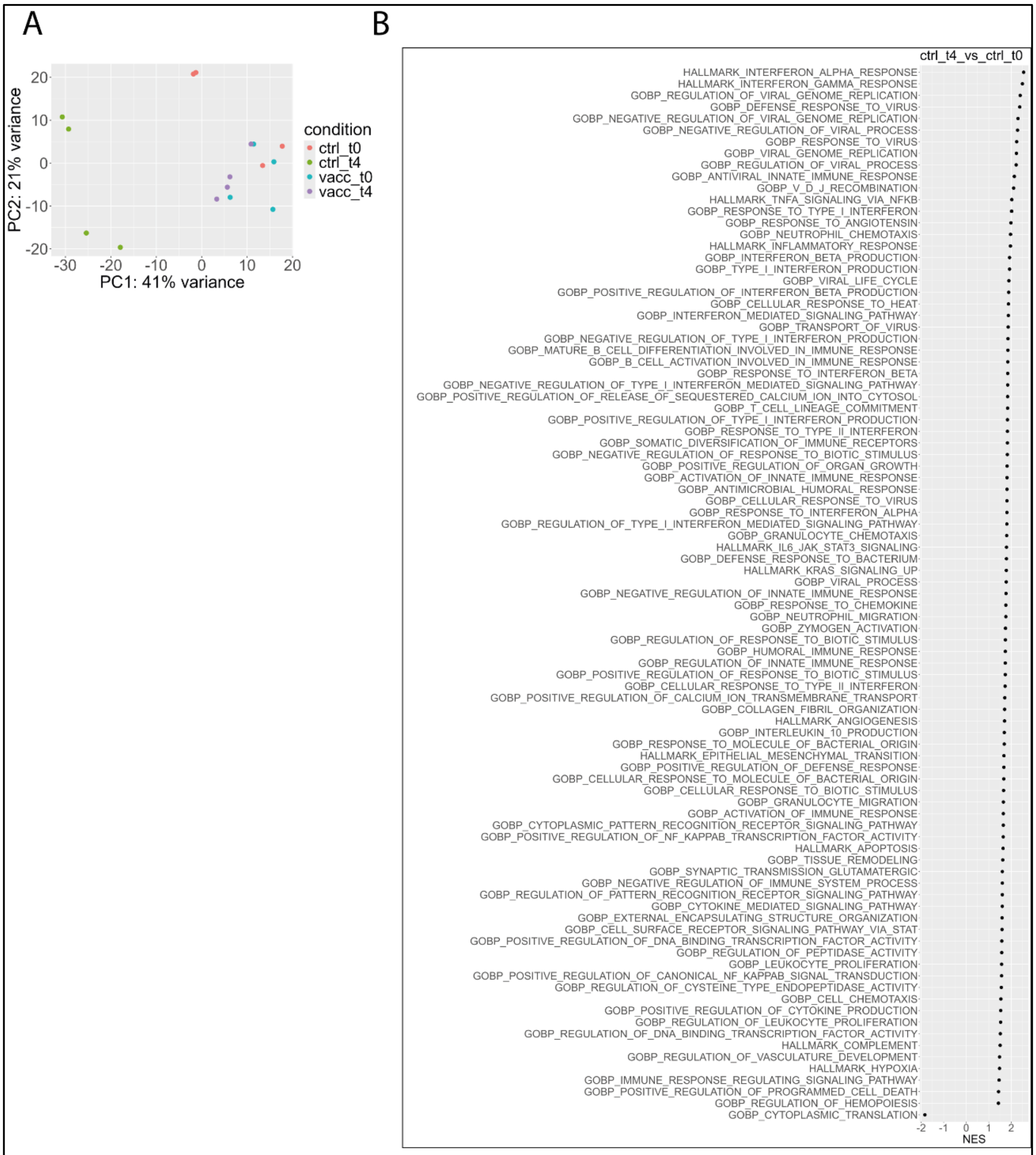

Figure S5. Transcriptomic profiling of PBMCs using RNA-seq reveals condition-dependent clustering and immune-related pathway activation. (A) Principal Component Analysis (PCA) of global gene expression from PBMCs of all experimental groups. Axes represent PC1 and PC2. (B) Gene Set Enrichment Analysis (GSEA) of PBMCs from unvaccinated animals comparing ctrl_t4 vs ctrl_t0, showing significantly enriched biological pathways (adjusted p-value < 0.05), ranked by Normalized Enrichment Score (NES).
